# Epistasis Detection and Modeling for Genomic Selection in Cowpea (*Vigna unguiculata* L. Walp.)

**DOI:** 10.3389/fgene.2019.00677

**Published:** 2019-07-30

**Authors:** Marcus O. Olatoye, Zhenbin Hu, Peter O. Aikpokpodion

**Affiliations:** ^1^Department of Crop Sciences, University of Illinois, Urbana-Champaign, IL, United States; ^2^Department of Agronomy, Kansas State University, Manhattan, KS, United States; ^3^Department of Genetics and Biotechnology, University of Calabar, Calabar, Nigeria

**Keywords:** cowpea, genetic architecture, epistasis, QTL, genomic-enabled breeding, genomic selection, flowering time, photoperiod

## Abstract

Genetic architecture reflects the pattern of effects and interaction of genes underlying phenotypic variation. Most mapping and breeding approaches generally consider the additive part of variation but offer limited knowledge on the benefits of epistasis which explains in part the variation observed in traits. In this study, the cowpea multiparent advanced generation inter-cross (MAGIC) population was used to characterize the epistatic genetic architecture of flowering time, maturity, and seed size. In addition, consideration for epistatic genetic architecture in genomic-enabled breeding (GEB) was investigated using parametric, semi-parametric, and non-parametric genomic selection (GS) models. Our results showed that large and moderate effect–sized two-way epistatic interactions underlie the traits examined. Flowering time QTL colocalized with cowpea putative orthologs of *Arabidopsis thaliana* and *Glycine max* genes like *PHYTOCLOCK1* (*PCL1* [Vigun11g157600]) and *PHYTOCHROME A* (*PHY A* [Vigun01g205500]). Flowering time adaptation to long and short photoperiod was found to be controlled by distinct and common main and epistatic loci. Parametric and semi-parametric GS models outperformed non-parametric GS model, while using known quantitative trait nucleotide(s) (QTNs) as fixed effects improved prediction accuracy when traits were controlled by large effect loci. In general, our study demonstrated that prior understanding of the genetic architecture of a trait can help make informed decisions in GEB.

## Introduction

Asymmetric transgressive variation in quantitative traits is usually controlled by non-additive gene interaction known as epistasis (Rieseberg et al., 1999). Epistasis has been defined as the interaction of alleles at multiple loci (Mathew et al., 2018). The joint effect of alleles at these loci may be lower or higher than the total effects of the loci (Johnson, 2008). In selfing species, epistasis is common due to high level of homozygosity (Volis et al., 2010) and epistatic interactions have been found among loci underlying flowering time in barley (Mathew et al., 2018), rice (Chen et al., 2015; Chen et al., 2018b), and sorghum (Li et al., 2018a). Although, theoretical models and empirical studies involving simulations have suggested the significant role for epistasis in breeding (Melchinger et al., 2007; Volis et al., 2010; Messina et al., 2011; Howard et al., 2014), empirical evidence from practical breeding are limited. In addition, most of the current statistical models cannot efficiently characterize or account for epistasis (Mackay, 2001; Moore and Williams, 2009; Sun et al., 2012; Mathew et al., 2018). Common quantitative traits mapping approaches are often single-locus analysis techniques. These techniques focus on the additive contribution of genomic loci (Barton and Keightley, 2002), which only explains a fraction of the genetic variation which can lead to missing heritability.

Regardless of the limitations of genomic mapping approaches, characterization of the genetic basis of complex agronomic traits has been beneficial for breeding purposes. For example, markers tagging quantitative trait loci (QTL) have been used in marker-assisted selection (MAS) in breeding programs (Zhang et al., 2003; Pan et al., 2006; Saghai Maroof et al., 2008; Foolad and Panthee, 2012; Massman et al., 2013; Mohamed et al., 2014; Zhao et al., 2014). However, the efficiency of QTL-based MAS approach in breeding is limited. First, the small sample size of bi-parental populations where QTL is detected often results in overestimation of the respective QTL effect sizes, a phenomenon known as Beavis effect (Utz et al., 2000; Xu, 2003; King and Long, 2017). Second, linkage mapping is limited in power to detect small effect loci; thus, only the available large effect loci are used for MAS (Ben-Ari and Lavi, 2012). Third, genetic diversity is limited to the two parents forming the bi-parental population; thus, QTL may not reflect the entire variation responsible for the trait and may not be transferable to other genetic backgrounds (Xu et al., 2017). Multi-parental populations as nested association mapping (NAM) and multiple advanced generation intercross (MAGIC) offer increased power, resolution, reliable estimate of QTL effects, and increased diversity than bi-parentals. Additionally, the MAGIC mapping population presents greater genetic diversity than bi-parentals to identify higher-order epistatic interactions (Mathew et al., 2018).

Notably, MAS is more efficient with traits controlled by few genomic loci than polygenic traits (Bernardo, 2008). In contrast, genomic selection (GS) that employs genome wide markers has been found to be more suited for complex traits, and also having higher response to selection than MAS (Bernardo and Yu, 2007; Wong and Bernardo, 2008; Cerrudo et al., 2018). In GS, a set of genotyped and phenotyped individuals are first used to train a model that estimates the genomic estimated breeding values (GEBVs) of un-phenotyped but genotyped individuals (Jannink et al., 2010). GS models often vary in performance with the genetic architecture of traits. Parametric GS models are known to capture additive genetic effects but are not efficient with epistatic effects due to the computational burden of high-order interactions (Moore and Williams, 2009; Howard et al., 2014). Parametric GS models with incorporated kernels (marker based relationship matrix) for epistasis have recently been developed (Covarrubias-Pazaran, 2016). Semi-parametric and non-parametric GS models capturing epistatic interactions have been developed and implemented in plant breeding (Gianola et al., 2006; Gianola and de los Campos, 2008; De Los Campos et al., 2010). Semi-parametric models as reproducing Kernel Hilbert space (RKHS) reduces parametric space dimensions to efficiently capture epistatic interactions among markers (Jiang and Reif, 2015; de Oliveira Couto et al., 2017). Using simulated data, Howard et al. (2014) showed that semi-parametric and non-parametric GS models can improve prediction accuracies under epistatic genetic architectures. In summary, different models may fit different genetic architectures. In general, GS has been widely studied and applied to major crop species including both cereals and legumes while its applications in orphan crop species has gained increased attention in recent times.

Cowpea (*Vigna unguiculata* L. Walp) is a widely adapted warm-season orphan herbaceous leguminous annual crop and an important source of protein in developing countries (Muchero et al., 2009; Varshney et al., 2012; Boukar et al., 2018; Huynh et al., 2018). Due to its flexibility as a “hungry season crop” (Langyintuo et al., 2003), cowpea is part of the rural families’ coping strategies to mitigate the effect of changing climatic conditions. Cowpea’s nitrogen fixing and drought tolerance capabilities make it a valuable crop for low-input and smallholder farming systems (Hall et al., 2003; Boukar et al., 2018). Breeding efforts using classical approaches have been made to improve cowpea’s tolerance to both biotic (disease and pest) and abiotic (drought and heat) stressors (Hall et al., 2003; Hall, 2004). Advances in applications of next-generation sequencing (NGS) and development of genomic resources (consensus map, draft genome, and multi-parent population) in cowpea have provided the opportunity for the exploration for GEB (Muchero et al., 2009; Boukar et al., 2018; Huynh et al., 2018). MAS and GS have improved genetic gain in soybean (*Glycine max*) (Jarquin et al., 2016; Kurek, 2018; Matei et al., 2018), common bean (*Phaseolus vulgaris*) (Schneider et al., 1997; Yu et al., 2000; Wen et al., 2019), chickpea (Roorkiwal et al., 2016; Li et al., 2018b), pigeonpea (Varshney et al., 2010; Pazhamala et al., 2015), and lentil (Haile et al., 2019). However, cowpea still lags behind major legumes in the area of GEB applications. GEB has the potential to expedite cowpea breeding to ensure food security in developing countries where national breeding programs still depend on labor-intensive and time-consuming classical breeding approaches.

In this study, we used the cowpea MAGIC population to first characterize the genetic architecture (main effect and epistatic effect loci) of flowering time, maturity, and seed size, and second, to evaluate considerations for genetic architecture in genomic-enabled breeding using parametric, semi-parametric, and non-parametric GS models and MAS. Our results showed that flowering time and maturity under short day are both controlled by moderate effect loci, while flowering time under long day and seed size are controlled by large and moderate effect loci. Also, accounting for large effect loci as fixed effects in parametric GS model improved prediction accuracy.

## Experimental Procedures

### Plant Genetic Resource and Phenotypic Evaluation

This study was performed using publicly available cowpea MAGIC population’s phenotypic and genotypic data (Huynh et al., 2018). The MAGIC population was derived from an intercross between eight founders. The F_1_s were derived from eight-way intercross between the founders and were subsequently selfed through single-seed descent for eight generations. The F_8_ RILs were later genotyped with 51,128 SNPs using the Illumina Cowpea Consortium Array. A core set of 305 MAGIC RILs were selected and phenotyped (Huynh et al., 2018). The RILs were evaluated under two irrigation regimes.

In this study, the flowering time (FLT), maturity (MAT), and seed size (SS) data were analyzed for environment-by-environment correlations and best linear unbiased predictions (BLUPs). The traits analyzed in this study are: FTFILD (FLT under full irrigation and long day), FTRILD (FLT under restricted irrigation and long day), FTFISD (FLT under full irrigation and short day), FTRISD (FLT under restricted irrigation and short day), FLT_BLUP (BLUP of FLT across environments), MFISD (MAT under full irrigation and short day), MRISD (MAT under restricted irrigation and short day), MAT_BLUP (BLUP of MAT across environments), SSFISD (SS under full irrigation and short day), SSRISD (SS under restricted irrigation and short day), and SS_BLUP(BLUP of SS across environments). In addition, using both genomic and phenotypic data, narrow sense heritability was estimated using *rrBLUP* package in R (Endelman, 2011).

### QTL and Epistasis Mapping

QTL mapping was performed for all traits using the stepwise regression model implemented in TASSEL 5.0 standalone version (Bradbury et al., 2007). The approach implements both forward inclusion and backward elimination steps. The model accounts for major effect loci and reduces collinearity among markers. The model was designed for multi-parental populations, and no family term was used in the model since MAGIC population development involved several steps of intercross that reshuffles the genome and minimizes phenotype-genotype covariance. A total of 32,130 SNPs across 305 RILs were used in the analysis. A permutation of 1,000 was used in the analysis.

To characterize the epistatic genetic architecture underlying FLT, MAT, and SS, the Stepwise Procedure for constructing an Additive and Epistatic Multi-Locus model (SPAEML; Chen et al., 2018a) epistasis pipeline implemented in TASSEL 5.0 was used to perform epistasis mapping for phenotypic traits (FTFILD, FTRILD, FTFISD, FTRISD, FT_BLUP, MFISD, MRISD, MT_BLUP, SSFISD, SSRISD, and SS_BLUP). One critical advantage of SPAEML that led to its consideration for this study is its ability to correctly distinguish between additive and epistatic loci. SPAEML source code is available at https://bitbucket.org/wdmetcalf/tassel-5-threaded-model-fitter. The minor allele frequency of each marker was estimated using a custom R script from http://evachan.org/rscripts.html. The additive effect of the marker was estimated as the difference between the mean phenotypic value of two homozygous classes of the alleles of a marker divided by two. The proportion of phenotypic variation explained (PVE) by each marker was estimated by multiplying the *R^2^* obtained from fitting a regression between the marker and the trait of interest by 100. The regression model for estimating PVE is:

[1] $$y_{ij}=\mu +\gamma_{\text{i}}+\varepsilon_{\text{ij}}$$

where *y_ij_* is the phenotype, *μ* is the overall mean, γ_i_ is the term for associated marker/SNP, and ε_ij_ is the residual term. This was implemented using the *lm* function in R.

A set of *a priori* genes (*n* = 100; **Data S1**) was put together from *Arabidopsis thaliana* and *G. max* FLT and SS genes obtained from literature and https://www.mpipz.mpg.de/14637/Arabidopsis_flowering_genes. The cowpea orthologs of these genes were obtained by blasting the *A. thaliana* and *G. max* sequence of the *a priori* genes on the new *Vigna* genome assembly *v.1* on Phytozome (Goodstein et al., 2012). The corresponding cowpea gene with the highest score was selected as a putative ortholog. Colocalizations between the cowpea putative orthologs and associated markers were identified using a custom R script. Only significant marker and *a priori* genes at the same genetic position were reported.

### Marker-Assisted Selection Pipeline

In order to evaluate the performance of MAS in cowpea, a custom pipeline was developed in R. Using subbagging approach, 80% of the 305 RILs randomly sampled without replacement was used as the training population, followed by performing a multi-locus GWAS (multi-locus mixed model, MLMM) (Segura et al., 2012) on both genomic and phenotypic data of the training population. The MLMM approach implements stepwise regression involving both forward and backward regressions. This model accounts for major effect loci and reduces the effect of allelic heterogeneity. A K-only model that accounts for a random polygenic term (kinship relationship matrix) was used in the MLMM model. No term for population structure was used in the model since MAGIC population development involved several steps of intercross that reshuffles the genome and minimizes phenotype-genotype covariance. A total of 32,130 SNPs across 305 RILs were used in the GWAS analysis and coded as −1 and 1 for homozygous markers/SNPs and 0 for heterozygous SNPs. Bonferroni correction with *α* = 0.05 was used to determine the cut-off threshold for each trait association (α/total number of markers = 1.6 e-06).

[2] y=Xβ+S∝+Zu+e

where *y* is the vector of phenotypic data, *β* is a vector of fixed effects other than SNPs, ∝ is the vector of SNP effects, u is a vector of polygenic background effects, and e is the vector of residual effects. X, S, and Z are incident matrices of 1s and 0s relating y to *β*, ∝, and u (Yu et al., 2006).

Afterwards, the top three most significant associations were then selected from the genomic data of the training population to train a regression model by fitting the SNPs as predictors in a regression model with the phenotypic information as the response variable. This training model was later used alongside the *predict* function in R to predict the phenotypic information of the validation population (20% that remained after sub-setting the training population). The prediction accuracy of MAS was obtained as the correlation between this predicted phenotypic information and the observed phenotypic information for the validation data.

### Genomic Selection Pipeline

In order to evaluate the performance of using known marker/SNP as fixed effects in GS models and to compare the performance of parametric, semi-parametric, and non-parametric GS models, a custom GS pipeline was developed in R. The GS pipeline was made up of four GS models, which were named as FxRRBLUP (ridge regression BLUP where markers were fitted as both fixed and random effects; parametric), RRBLUP (RRBLUP where markers were only fitted as random effects; parametric), reproducing Kernel Hilbert space (RKHS; semi-parametric), and support vector regression (SVR; non-parametric). First, using subagging approach, 80% of the RILs were randomly sampled without replacement (training population) followed by running MLMM GWAS and selecting the three most significant associations, which were used as fixed effects in the FxRRBLUP. These three SNPs were removed from the rest of SNPs that were fitted as random effects in the FxRRBLUP model. Using a high number of SNPs as fixed effects have been found to increase bias (Rice and Lipka, 2019), as a result, three QTNs were fitted as fixed effects. The RRBLUP, RKHS, and SVR models were fitted simultaneously in the same cycle as FxRRBLUP to ensure unbiased comparison of GS models. Likewise, in order to ensure unbiased comparison between GS and MAS approaches, similar seed numbers were used for the subagging sampling of training populations across 100 cycles for GS and MAS. The validation set was composed of the remaining 20% of the RILs after sampling the 80% (training set). Prediction accuracy in GS was estimated as the Pearson correlation between measured phenotype and GEBVs of the validation population. Also, for FLT, each environment was used as a training population to predict the other three environments.

#### Ridge Regression BLUP (RRBLUP)

The two RRBLUP models (with and without fixed-effect term) can be described as;

[3] $$y=\mu +\sum_{m=1}^{p}Z_{m}u_{m}+e$$

[4] $$y=\mu +\sum_{k=1}^{q}X_{k}\alpha_{k}+\sum_{m=1}^{p}Z_{m}u_{m}+e$$

where **y** is the vector (*n* x 1) of observations (phenotypic data), *μ* is the vector of the general mean, *q* is the number of selected significant associated markers (*q* = 3), ***X_k_*** is the *k* ^th^ column of the design matrix ***X***, *α* is the fixed additive effect associated with markers *k … q*, *u* random effects term, with *E*(*u_m_*) = 0, $Var(u_{m})=\sigma_{u_{m}}^{2}$ (variance of marker effect), *p* is the marker number (*p* > *n*), ***Z*** *_m_* is the *m* ^th^ column of the design matrix ***Z***, and *u* is the vector of random marker effects associated with markers *m … p*. In the model, *u* random effects term, with *E*(*u_m_*) = 0, $Var(u_{m})=\sigma_{u_{m}}^{2}$ (variance of marker effect), *Var*(**e**) = *σ* ^2^ (residual variance), *Cov*(u, **e**) = 0, and the ridge parameter *λ* equals σe2σu2 (Meuwissen et al., 2001; Endelman, 2011; Howard et al., 2014). In this study, RRBLUP with and without fixed effects were implemented using the *mixed.solve* function in *rrBLUP* R package (Endelman, 2011).

#### Reproducing Kernel Hilbert Space (RKHS)

Semi-parametric models are known to capture interactions among loci. The semi-parametric GS approach used in this study was implemented as Bayesian RKHS in *BLGR* package in R (Perez, 2014), and described as follows:

[5] y=1μ+u+ε

where ***y*** is the vector of phenotype, **1** is a vector of 1’s, *μ* is the mean, ***u*** is vector of random effects ∼MVN (**0**, $K_{h}\sigma_{u}^{2}$), and *ε* is the random residual vector ∼ MVN (**0**, ***I***$\sigma_{\varepsilon }^{2}$). In Bayesian RKHS, the priors *p*(ㅰ*μ*, ***u***, ε) are proportional to the product of density functions MVN (**0**, $K_{h}\sigma_{u}^{2}$) and MVN (**0**, ***I***$\sigma_{\varepsilon }^{2}$). The kernel entries matrix (**K*_h_***) with a Gaussian kernel uses the squared Euclidean distance between marker genotypes to estimate the degree of relatedness between individuals, and a smoothing parameter (*h*) multiplies each entry in **K*_h_*** by a constant. In the implementation of RKHS, a default smoothing parameter *h* of 0.5 was used alongside 1,000 burns and 2,500 iterations.

#### Support Vector Regression (SVR)

Support vector regression method (Vapnik, 1995; Maenhout et al., 2007; Long et al., 2011) was used to implement non-parametric GS approach in this study. The aim of the SVR method is to minimize prediction error by implementing models that minimizes large residuals (Long et al., 2011). Thus, it is also referred to as the “ε-intensive” method. It was implemented in this study using the normal radial function kernel (*rbfdot*) in the *ksvm* function of *kernlab* R package (Karatzoglou et al., 2004).

### Parameters Evaluated in GS and MAS

Additional parameters were estimated to further evaluate the performance of GS and MAS models. A regression model was fitted between observed phenotypic information and GEBV of the validation set to obtain both intercept and slope for both GS and MAS in each cycle of prediction. The estimates of the intercept and slope of the regression of the observed phenotypic information on GEBVs are valuable since their deviations from expected values can provide insight into deficiencies in the GS and MAS models (Daetwyler et al., 2013). The bias estimate (slope and intercept) signifies how the range of values in measured and predicted traits differ from each other. In addition, the coincidence index between the observed and GEBVs for both GS and MAS models was evaluated. The coincidence index (Fernandes et al., 2018) evaluates the proportion of individuals with highest trait values (20%) that overlapped between the measured phenotypes and predicted phenotypic trait values for the validation population.

## Results

### Phenotypic and Genotypic Variation in Cowpea

**Figure 1:**
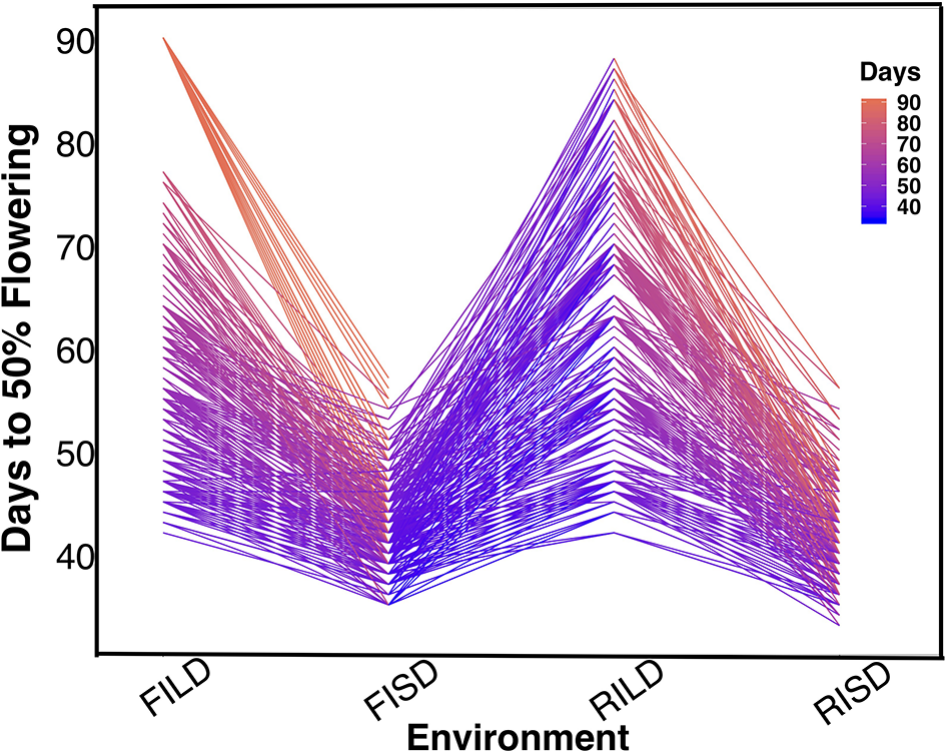
The reaction norm plot for flowering time variation under long-day and short-day periods. Evaluation environments are represented on the *x*-axis (full irrigation and long day [FILD], full irrigation and short day [FISD], restricted irrigation and long day [RILD], and restricted irrigation and short day [RISD]). The number of days to 50% flowering is represented on the *y*-axis.

Results showed variation between number of days to 50% flowering under long-day photoperiod and short-day photoperiod. Days to FLT were higher for RILs under long day than short day (**Figure 1**). Results showed positive high correlations between environments for each trait (**Tables S1** and **S2**). Furthermore, genomic heritability were moderate for the traits ranging between 0.41 under long-day photoperiod to 0.48 for FLT under short-day photoperiod, 0.21 under restricted irrigation to 0.30 under full irrigation for MAT, and 0.39 under restricted irrigation to 0.47 under full irrigation for SS (**Tables S1** and **S2**).

### Genetic Architecture of Traits

#### Main Effect QTL

The cowpea multi-parental advanced generation intercross (MAGIC) population facilitated the characterization of the genetic architecture of FLT, MAT, and SS. In this study, QTL associated with FLT, MAT, and SS were identified using stepwise regression analysis (**Table S3**, **Data S2**). Results showed that 32 QTL (22 unique) in total were associated with FLT traits (FT_BLUP [eight QTLs, explaining 73.2% of phenotypic variation (PV)], FTFILD [five QTL, explaining 66.2% of PV], FTRILD [five QTL explaining 48.6% of PV], FTFISD [eight QTL explaining 52.1% of PV], and FTRISD [six QTL explaining 43.9% of PV]). Each of the total QTL associated with FLT traits explained between 2 and 28% of the phenotypic variation. QTL qVu9:23.36, qVu9:24.77, and qVu9:22.65 (MAF = 0.29, 0.28, and 0.49) explained the largest proportion of variation (28%, 24%, and 19%) with additive effects of 7, 7, and 6 days, respectively. The minor allele frequency (MAF) of the FLT QTL ranges from 0.13 to 0.50. For MAT traits, 13 QTL (11 unique QTL) in total were identified with five QTL (explaining 35.9% of PV) for MAT_BLUP, four QTL (explaining 24.5% of PV) for MFISD, and four QTL (explaining 27.9% of PV) for MRISD. All MAT trait QTL explained between 4.5 to 10% of phenotypic variation and MAF ranges from 0.15 to 0.49.

**Figure 2:**
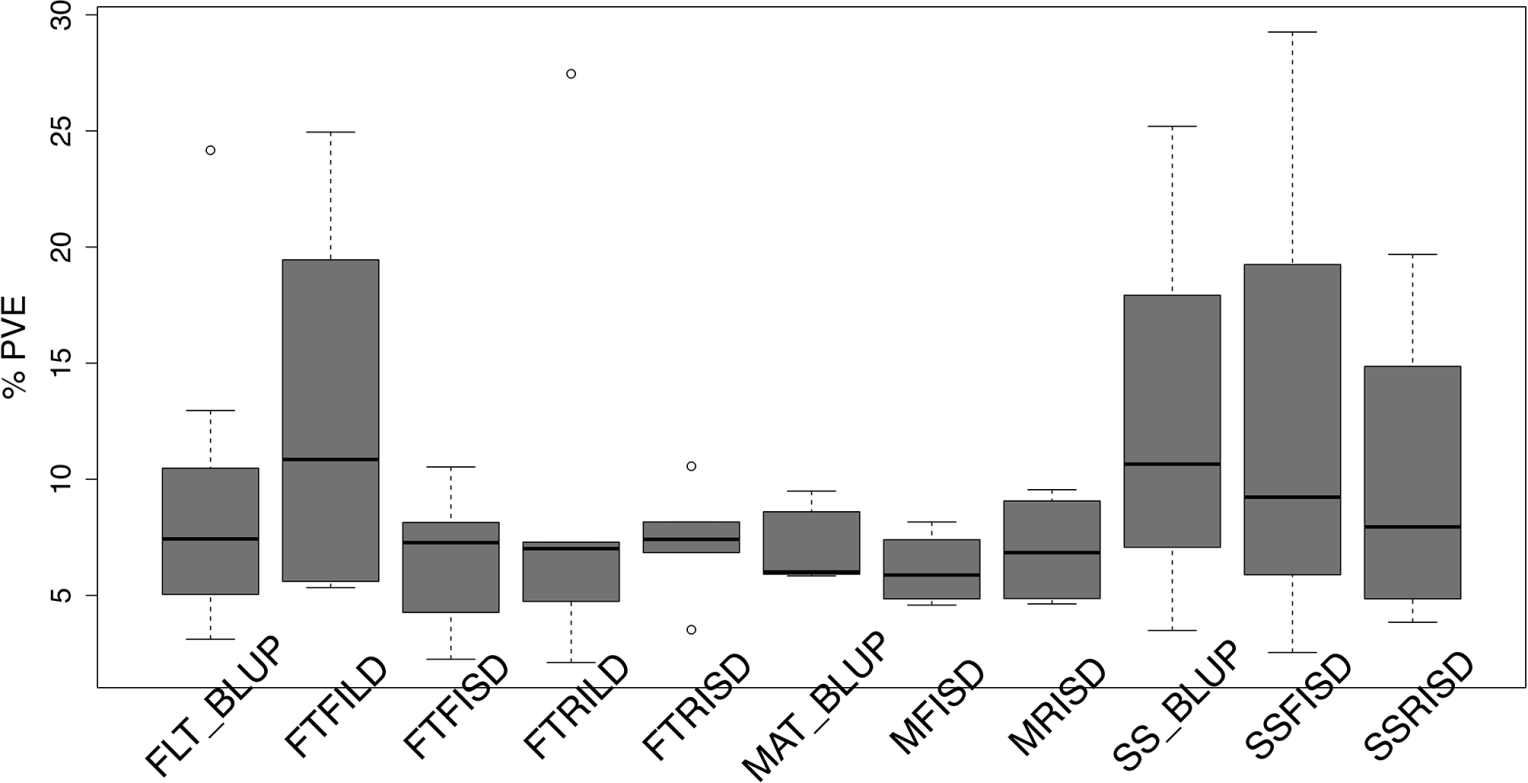
Distribution of effect size of quantitative traits loci (QTL) associated with traits in the cowpea MAGIC population. Box plots of the distribution of proportion of variation explained (PVE) by quantitative traits loci (QTL) associated with best linear unbiased predictions (BLUP) of flowering time across environments (FLT_BLUP), flowering time under full irrigation and long day (FTFILD), flowering time under restricted irrigation and long day (FTRILD), flowering time under full irrigation and short day (FTFISD), flowering time under restricted irrigation and short day (FTRISD), BLUP of maturity across environments (MAT_BLUP) maturity under full irrigation and short day (MFISD), maturity under restricted irrigation and short day (MRISD), BLUP of seed size across environments (SS_BLUP) seed size under full irrigation and short day (SSFISD), and seed size under restricted irrigation and short day (SSRISD).

Furthermore, for SS traits, 10 QTL (seven unique QTL) in total were identified with three QTL (explaining 39.3% of PV) for SS_BLUP, three QTL (explaining 41% of PV) for SSFISD, and four QTL (explaining 39.4% of PV) for SSRISD. QTL qVu8:74.21, qVu8:74.29, and qVu8:76.81 associated with SSFISD, SS_BLUP, and SSRISD explained the largest PV (29%, 25%, and 20%). All SS trait QTL explained between 3 and 29% of PV and with MAF range between 0.21 and 0.49. A pleiotropic QTL qVu8:74.21 (MAF = 0.24) was associated with both MRISD and SSRISD (explained 5% and 29% of PV, respectively). In summary, QTL effects range from small to large for all traits in this study (**Figure 2**).

#### Two-Way Epistatic Interaction QTL

Currently, there is limited knowledge about what role epistasis plays in phenotypic variation in cowpea. Our results identified epistatic loci underlying FLT, MAT, and SS (**Table S4**, **Data S3**). For FLT traits, there were 42 two-way epistatic interactions at 84 epistatic loci (only 65 loci were unique). Among these are; 20 epistatic loci for FLT_BLUP, 18 epistatic or FTFILD, 12 epistatic loci for FTRILD, 14 epistatic loci for FTFISD, and 20 epistatic loci for FTRISD. Some large effect loci were involved in epistatic interactions in FLT; examples include, QTL qVu9:25.39 (MAF = 0.28, FT_BLUP PVE = 23.5%, FTFILD PVE = 24.5%, FTRILD PVE = 26%) and QTL qVu9:3.46 (MAF = 0.35, FLT_BLUP PVE = 13.5%, FTRILD PVE = 14.1%). For MAT, there were 17 pairwise epistatic interactions across 34 loci (of which 30 were unique). Among the MAT QTL, qVu9:8.37 had the largest effect explaining ∼9% of the phenotypic variation. One epistatic interaction overlapped with both FTRISD, MRISD, and MT_BLUP (qVu2:48.05+ qVu9:8.37, MAF = 0.30, and 0.39, respectively). For SS, there were 13 interactions at 26 loci (19 were unique). Only one QTL (qVu8:74.29, MAF = 0.25) had interactions with multiple QTL. The largest effect epistatic QTL associated with the three SS traits (SS_BLUP, SSFISD, and SSRISD) is qVu8:74.29 (MAF0.25). Some QTL were found to overlap among main effect QTL and epistatic effect QTL for FLT (nine QTL), MAT (three QTL), and SS (three QTL) (**Figure S1**).

#### Main Effect and Epistatic QTL Colocalized with *A priori* Genes

**Figure 3:**
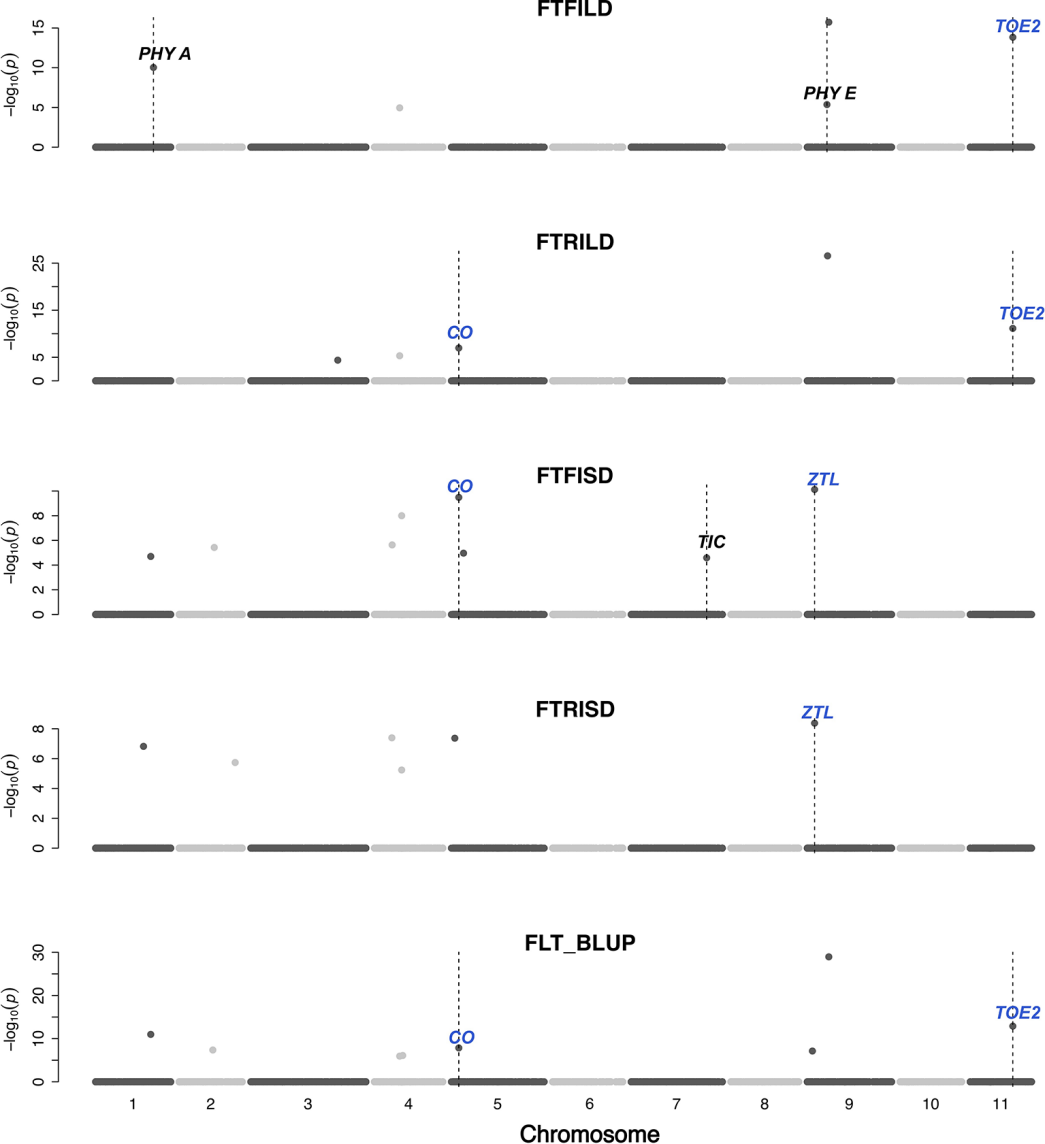
Main QTL plot for flowering time traits in the cowpea MAGIC population. QTL plots for flowering time under full irrigation and long day (FTFILD), flowering time under restricted irrigation and long day (FTRILD), flowering time under full irrigation and short day (FTFISD), flowering time under restricted irrigation and short day (FTRISD), and BLUPs of environments (FLT_BLUP). The chromosome numbers are located on the x-axis and the negative log of the P-values on the y-axis. The genetic position of the colocalization between QTL and *a priori* genes are indicated by broken vertical lines. The texts displayed on the vertical broken lines are the names of *a priori* genes (blue for genes associated with multiple environments or traits, and black for genes associated with single environments or trait).

**Figure 4:**
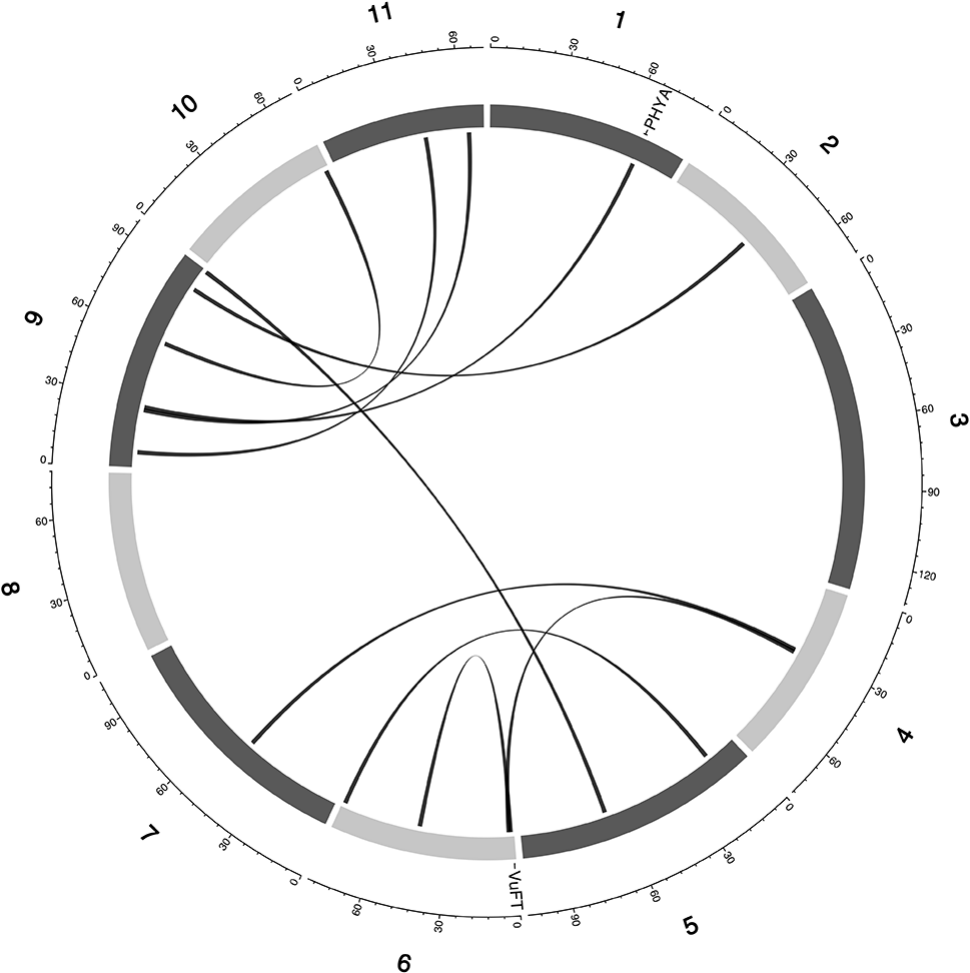
Epistatic QTL for FLT_BLUP for MAGIC population. Chromosomes are shown in shades of gray, two-way interacting loci are connected with black solid lines, and colocalized *a priori* genes are texts between chromosomes and genetic map.

**Figure 5:**
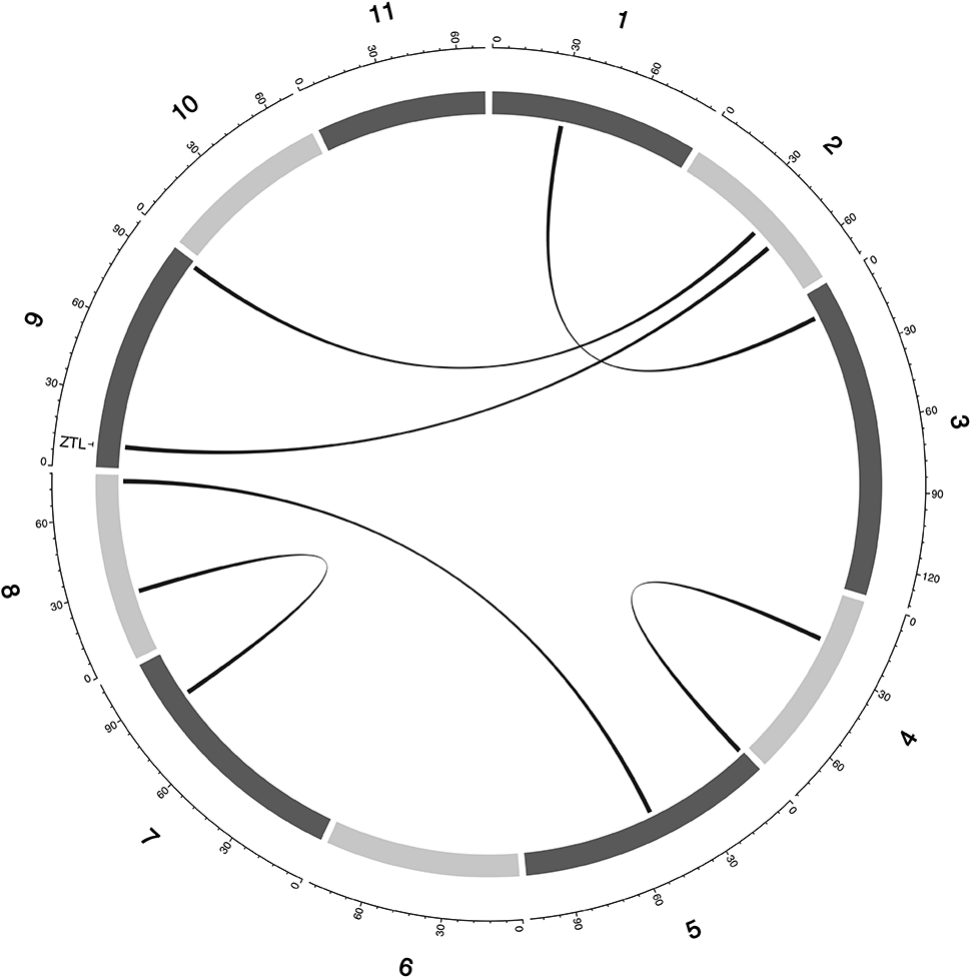
Epistatic QTL for MAT_BLUP in MAGIC population. Chromosomes are shown in shades of gray, two-way interacting loci are connected with black solid lines, and colocalized *a priori* genes are texts between chromosomes and genetic map.

**Figure 6:**
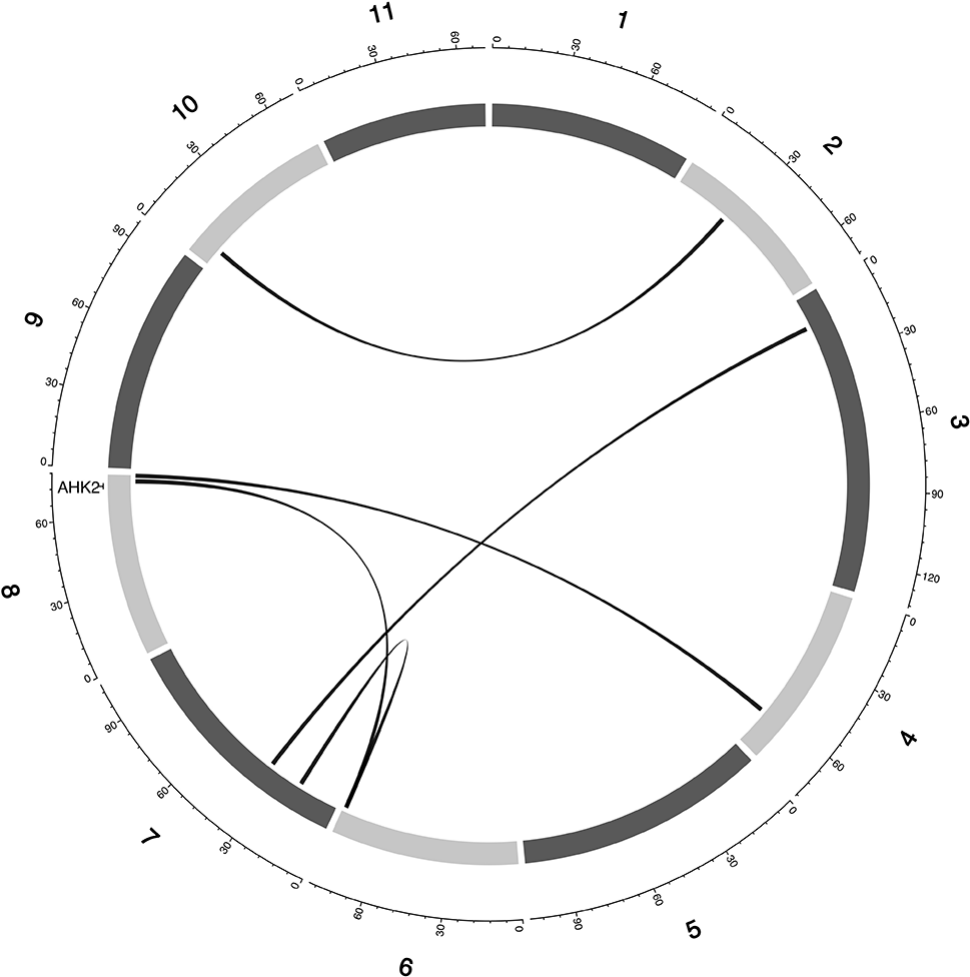
Epistatic QTL for MAT_BLUP in MAGIC population. Chromosomes are shown in shades of gray, two-way interacting loci are connected with black solid lines, and colocalized *a priori* genes are texts between chromosomes and genetic map.

Gene functions can be conserved across species (Huang et al., 2017). In this study, a set of *a priori* genes was compiled from both *A. thaliana* and *G. max*. Both main effect QTL and epistatic QTL colocalized with putative cowpea orthologs of *A. thaliana* and *G. max* FLT and SS genes (**Figures 3**–**6**, **Figures S2–S11**, **Data S4**) at the same genetic position. However, two genes (*TOE2* and *AHK2*) did not colocalize with the QTL at the same genetic position but were reported due to their proximity and biological relevance. A putative cowpea ortholog (Vigun09g050600) of *A. thaliana* circadian clock gene *phytochrome E* (*PHYE*; AT4G18130) (Aukerman and Sakai, 2003) colocalized with FTFILD QTL (qVu9:22.65; PVE = 19.5%; main effect QTL) at the same genetic position. Also, a putative cowpea ortholog (Vigun07g241700) of *A. thaliana* circadian clock gene *TIME FOR COFFEE (TIC*; AT3G22380) (Hall et al., 2003) colocalized at the same genetic position with FTFISD QTL (qVu7:86.92; PVE = 2.6%; main effect QTL). The cowpea FLT gene (*VuFT*; Vigun06g014600; CowpeaMine *v*.06) colocalized with an epistatic QTL (qVu6:0.68; PVE = 3.5%) associated with FLT_BLUP and FTRILD at the same genetic position. The cowpea ortholog (Vigun11g157600) of *A. thaliana* circadian clock gene *PHYTOCLOCK1* (*PCL1*; AT3G46640) (Hazen et al., 2005) colocalized with an epistatic QTL (qVu11:50.94; PVE = 8–10%) associated with both FTFILD and FTRILD at the same genetic position.

A putative cowpea ortholog (Vigun11g148700) of *A. thaliana* photoperiod gene *TARGET OF EAT2* (*TOE2*; AT5G60120) (Mathieu et al., 2009) was found at a proximity of 0.6cM from a QTL (qVu11:49.06; PVE = 7–11%; main effect QTL) associated with FTFILD, FTRILD, and FLT_BLUP. Some of the *a priori* genes colocalized with some QTL that are both main effect and epistatic QTL. For instance, the cowpea ortholog (Vigun01g205500) of *G. max* FLT gene *phytochrome A* (*PHYA*; Glyma19g41210) (Tardivel et al., 2014) colocalized with a FTFILD QTL (qVu1:66.57; PVE = 5.3%; both main effect and epistatic QTL) at the same genetic position (Data S4). Lastly, a putative cowpea ortholog (Vigun08g217000) of *A. thaliana histidine kinase2* gene (*AHK2*; AT5G35750) (Orozco-Arroyo et al., 2015) was found at a proximity of about 1–2cM from three QTL (qVu8:74.29, qVu8:74.21, qVu8:76.81; PVE = 25%, 29.3%, and 20%, respectively; main effect and epistatic QTL) associated with SS traits SS_BLUP, SSFISD, and SSRISD). In addition, some *a priori* genes were associated with multiple traits. The putative cowpea ortholog (Vigun05g024400) of *A. thaliana* circadian clock gene *CONSTANS* (*CO*; AT5G15840) (Wenkel et al., 2006) colocalized at the same genetic position with a QTL (qVu5:8.5; PVE = 6–8%; both main effect and epistatic QTL) associated with FLT and MAT traits (FLT_BLUP, FTFISD, FTRILD, FTRISD, MAT_BLUP, and MFISD). The putative cowpea ortholog (Vigun09g025800) of *A. thaliana* circadian clock gene *ZEITLUPE* (*ZTL*; AT5G57360) (Somers et al., 2000) colocalized at the same genetic position with a QTL (qVu9:8.37; PVE = 9–11%; both main effect and epistatic QTL) associated with FLT and MAT traits (FTFISD, FTRISD, and MRISD).

### GS and MAS for Flowering Time

**Figure 7:**
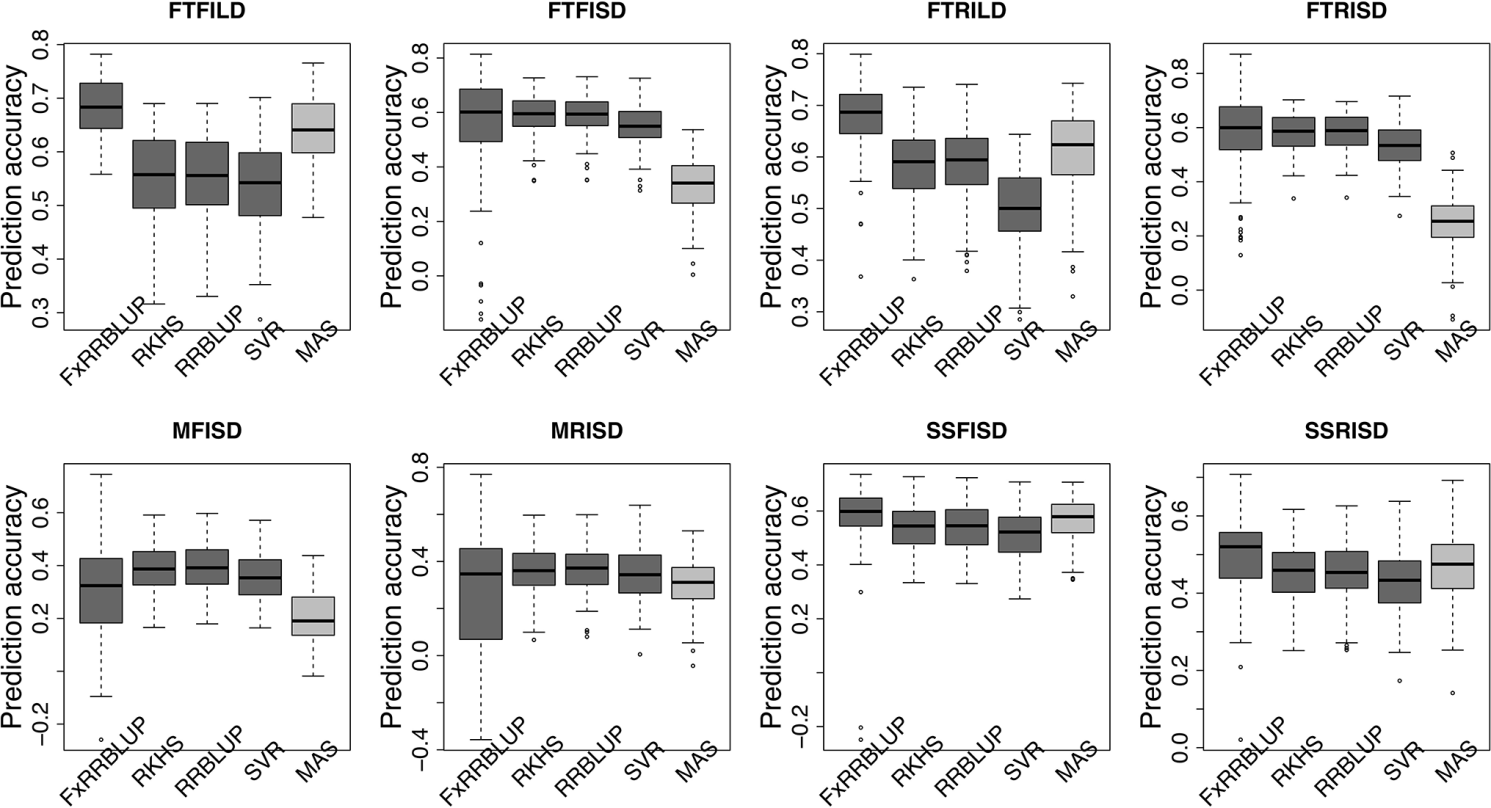
Comparison of prediction accuracy across GS and MAS models. Boxplots in each panel showed the distribution of prediction accuracy values across 100 cycles for FxRRBLUP (ridge regression best linear unbiased prediction: parametric model with fixed effects), RKHS (reproducing Kernel Hilbert space; semi-parametric model), RRBLUP (ridge regression best linear unbiased prediction: parametric model with no fixed effects), SVR (support vector regression: non-parametric model), and MAS (marker-assisted selection) for flowering time under full irrigation and long day (FTFILD), flowering time under restricted irrigation and long day (FTRILD), flowering time under full irrigation and short day (FTFISD), flowering time under restricted irrigation and short day (FTRISD), maturity under full irrigation and short day (MFISD), maturity under restricted irrigation and short day (MRISD), seed size under full irrigation and short day (SSFISD), and seed size under restricted irrigation and short day (SSRISD).

**Figure 8:**
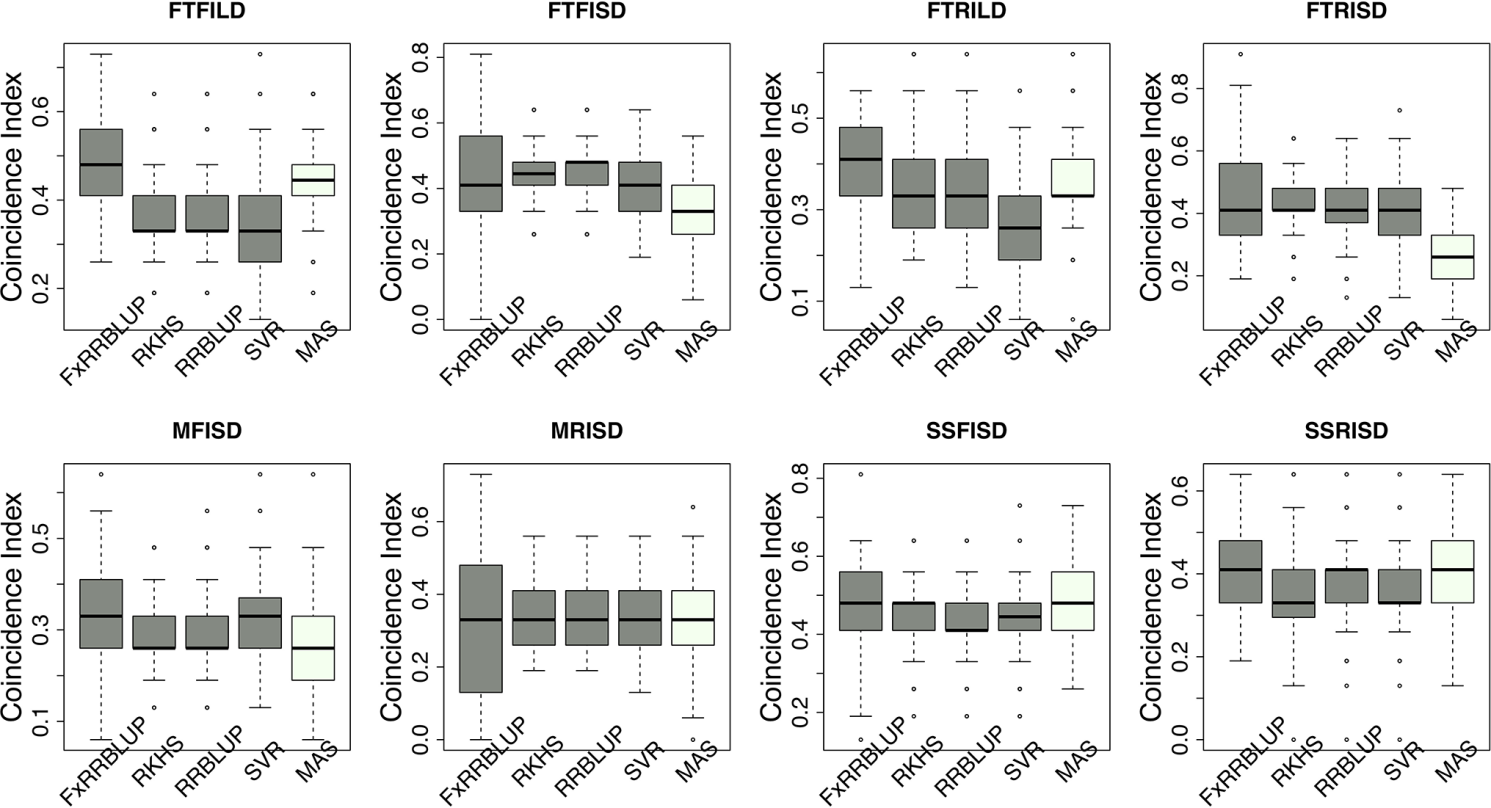
Comparison of coincidence index across GS and MAS models. Boxplots in each panel showed the distribution of coincidence index values across 100 cycles for FxRRBLUP (ridge regression best linear unbiased prediction: parametric model with fixed effects), RKHS (reproducing Kernel Hilbert space; semi-parametric model), RRBLUP (ridge regression best linear unbiased prediction: parametric model with no fixed effects), SVR (support vector regression: non-parametric model), and MAS (marker-assisted selection) for flowering time under full irrigation and long day (FTFILD), flowering time under restricted irrigation and long day (FTRILD), flowering time under full irrigation and short day (FTFISD), flowering time under restricted irrigation and short day (FTRISD), maturity under full irrigation and short day (MFISD), maturity under restricted irrigation and short day (MRISD), seed size under full irrigation and short day (SSFISD), and seed size under restricted irrigation and short day (SSRISD).

Prior knowledge about the genetic architecture of a trait can help make informed decisions in breeding. Comparing the performance of GS and MAS models for FLT within each daylength results showed that, under long day length (FTFILD and FTRILD), FxRRBLUP (mean prediction accuracy [mPA] = 0.68, 0.68; mean coincidence index [mCI] = 0.49, 0.40) and MAS (mPA = 0.64, 0.61; mCI = 0.45, 0.37) outperformed RRBLUP (mPA = 0.55, 0.58; mCI = 0.37, 0.35), RKHS (mPA = 0.55, 0.58; mCI = 0.37, 0.36), and SVR (mPA = 0.54, 0.50; mCI = 0.35, 0.28) (**Figures 7** and **8**, **Tables S3** and **4**). For FLT under long day, coincidence index values were higher under full irrigation than under restricted irrigation. For FLT under short day (FTFISD and FTRISD), all GS models outperformed MAS (mPA = 0.33, 0.25; mCI = 0.30, 0.26). Among the GS models, RKHS and RRBLUP had the highest prediction accuracies. However, the coincidence index of FxRRBLUP was higher than the rest of the GS models for FTRISD. In general, the mean of the slope and intercept for the GS models except SVR were usually close to the expected (1 and 0) (**Figures S12–S13**). MAS also deviated away from the expected slope and intercept (1 and 0) more than the FxRRBLUP, RKHS, and RRBLUP for FTRISD (**Figures S12–S13**). To evaluate the effect of photoperiod and irrigation regime on the performance of training population, each environment (day length and irrigation regime combination) was used as a training population to predict the rest in a di-allele manner. Results showed that prediction accuracy between environments in the same photoperiod was higher than environments in different photoperiod (**Figure S14**). Also, when training populations were under full irrigation, their prediction accuracies were higher than when training populations were under restricted irrigation (**Figure S14**). For FT_BLUP, GS models outperformed MAS except SVR which had the same mPA (0.59) as MAS while FxRRBLUP had the highest mPA and mCI among the GS models (**Figures S15** and **16**). Overall, **Table S7** showed that FxRRBLUP had the best performance in six out of the eight traits by environment combination.

### GS and MAS for Maturity and Seed Size

For MAT (MT_BLUP, MFISD, and MRISD), RKHS and RRBLUP had better performance (**Figures 7** and **8**; **Tables S4** and **S5**) than the rest of the models including MAS. All models deviated from the expected slope and intercept estimates, but RRBLUP had the least deviation for MRISD. For SS, FxRRBLUP had the best performance followed by MAS compared to the rest of the GS models (RKHS, RRBLUP, and SVR) (**Figures 7** and **8**; **Tables S5** and **S6**). GS and MAS models had varying levels of deviation from the expected estimates of slope and intercept. RKHS and RRBLUP were closer to the expected than FxRRBLUP and MAS (**Figures S12–S13**) while SVR had the highest deviation.

## Discussion

### Epistasis Plays Important Roles in Determining the Genetic Architecture of Agronomic Traits in Cowpea

Multi-parental populations have demonstrated ability to facilitate robust characterization of genetic architecture in terms of genetic effect size, pleiotropy, and epistasis (Buckler et al., 2009; Brown et al., 2011; Peiffer et al., 2014; Bouchet et al., 2017; Mathew et al., 2018). Using the cowpea MAGIC population, this study showed that both additive main QTL and additive × additive epistatic QTL with large and (or) moderate effects underlie FLT, MAT, and SS in cowpea. Although we identified two-way epistatic interactions, results showed that some loci were involved in interactions with more than one independent loci (**Figures 4** and **5** and **Figures S4–11**). This implies the possibility of three-way epistatic interactions underlying some of the traits. Our inability to identify and discuss three-way epistatic interactions is due to the mapping approach used, which only mapped two-way epistatic interactions. Three-way epistatic interactions have been found to underlie FLT in the selfing crop specie barley (Mathew et al., 2018). Furthermore, overlaps between main and epistatic loci (**Figure S2**) indicate these to be main effect loci that are involved in epistatic interactions with other loci. However, one caveat that may also be responsible for some of the QTL among the overlaps is the false positive rate of SPEAML. The SPEAML software used for epistasis mapping showed high false positive rate with a sample size of 300 individuals (Chen et al., 2018a). It is possible that some of the overlapped QTL are main QTL that were miscategorized as epistatic loci by SPEAML since our cowpea MAGIC population had 305 RILs.

### Distinct and Common Genetic Regulators Underlie Flowering Time

FLT is an important adaptive trait in breeding. Photoperiod impacted days to FLT as observed from the reaction norm plot for cowpea MAGIC FLT data which showed drastic reductions in days to flowering for RILs under short day compared to long days (**Figure 1**). Our mapping results (main effect and epistatic) showed that both unique and common loci underlie FLT variation under long and short photoperiod (**Figure 1**; **Figures S4–S8**). Epistatic loci underlie FLT in both selfing (Komeda, 2004; Juenger et al., 2005; Huang et al., 2013; Chen et al., 2018b; Li et al., 2018a; Mathew et al., 2018) and outcrossing (Buckler et al., 2009; Durand et al., 2012) species. In addition, the effect size of FLT loci differs between selfing and out crossing species as QTL effect sizes are large in the former (Lin et al., 1995; Maurer et al., 2015) and small in the later (Buckler et al., 2009). In the present study, the large effects (up to 25% PVE and additive effect of 7 days) of FLT loci were only identified under long-day photoperiod and not under short-day photoperiod (**Figure 2**, **Tables S3** and **S4**). The loci detected under short-day photoperiod were of moderate effects (PVE = 1–10% and maximum additive effect size of 2 days). The large effect size attributed to some of the loci that are unique to FLT adaptation under long photoperiod suggests the possible effect of recent selection ate these loci (Orr, 1998; Orr, 1999; Brown et al., 2011; Dittmar et al., 2016).

Conserved genetic pathways often underlie traits in plant species (Liu et al., 2013; Huang et al., 2017). Examination of colocalizations between *a priori* genes and QTL in this study identified putative cowpea orthologs of *A. thaliana* and *G. max* FLT that may underlie phenotypic variation in cowpea. FLT is affected by photoperiodicity and regulated by a network of genes (Sasaki et al., 2018) involved in floral initiation, circadian clock regulation, and photoreception (Lin, 2002). In addition, certain *a priori* genes were unique to either FLT under long day or short day. For instance, cowpea putative orthologs of photoreceptors (*PHY A* [Vigun01g205500] and *PHY E* [Vigun09g050600]) and circadian clock gene *PHYTOCLOCK1* (*PCL1* [Vigun11g157600]) colocalized with only QTL associated with FLT under long day, while cowpea putative orthologs of circadian clock genes (*Time for Coffee* [*TIC* (Vigun07g241700)] and *Zeitlupe* [*ZTL*]) colocalized with only QTL associated with FLT under short day. However, the cowpea putative ortholog of photoperiod gene *CONSTANS* (*CO* [Vigun05g024400]) colocalized with QTL associated with FLT under both long and short days. Thus, our study suggests that distinct and common genetic regulators control FLT adaptation to both long- and short-day photoperiod in cowpea. Further studies utilizing functional approaches will be helpful to decipher gene regulation patterns under both long- and short-photoperiod in cowpea.

### Genetic Basis of Maturity and Seed Size

In this study, the genetic basis of MAT and SS were evaluated under short-day photoperiod only. Our study demonstrated that MAT under short day is controlled by moderate and small effect main and epistatic loci. MAT QTL were found to colocalize with cowpea putative orthologs of *Arabidopsis* circadian clock and photoperiod (*ZTL* [*ZEITLUPE*], *CO* [*CONSTANS*]) genes. One pleiotropic QTL (qVu9:8.37 colocalized with *ZTL* [*ZEITLUPE*]) was found to be associated with both MAT and FLT under restricted irrigation and short-day photoperiod. Pleiotropic QTL between MAT and FLT were also reported in soybean (Kong et al., 2018). This suggest a possible genetic basis for the positive relationship found between MAT and FLT in prior studies (Huynh et al., 2018; Owusu et al., 2018). A major large effect locus explaining up to 29% of the phenotypic variation was found to be associated with SS. This QTL was found at about 2cM from the cowpea ortholog of *Arabidopsis AHK2* SS gene. Further studies, using mapping panels with more diverse founders and more *a priori* genes will be required to identify further genes underlying natural variations in MAT and SS in cowpea.

### Genetic Architecture Influenced GS and MAS Performance

GS models differ in their efficiency to capture complex cryptic interactions among genetic markers (de Oliveira Couto et al., 2017). The traits evaluated in this study are controlled by both main effect and epistatic loci. In this study, comparison among the GS models showed that parametric and semi-parametric GS models outperformed non-parametric GS model for all traits. SVR, a non-parametric model, had the least prediction accuracy and coincidence index and also had the highest bias (**Figures S12** and **S13**). Previous studies have shown that semi-parametric and non-parametric models increased prediction accuracy under epistatic genetic architecture (Howard et al., 2014; Jacquin et al., 2016). In this study, none of semi-parametric and non-parametric models outperformed parametric models (**Figures 6** and **7**). Some of the studies comparing the performance of parametric, semi-parametric, and non-parametric GS models were based on simulations of traits controlled solely by epistatic genetic architectures. Therefore, the performance of the models under simulated combined genetic effects (additive + epistasis) is not well understood. The comparable performance of RKHS to RRBLUP (parametric model) in this study in terms of prediction accuracy, coincidence index, and bias estimates attests to RKHS ability to capture both additive and epistatic interactions (Gianola et al., 2006; Gianola and Van Kaam, 2008; De Los Campos et al., 2010; Gota and Gianola, 2014) for both prediction accuracy and selection of top performing lines. The performance of GS models is often indistinguishable, and RRBLUP has been recommended as an efficient parametric GS model (Heslot et al., 2012; Lipka et al., 2015). SVR had the worst performance with extremely high bias estimates.

Understanding the genetic architecture of agronomic traits can help improve accuracy of genomic predictions (Hayes et al., 2010; Swami, 2010). Our study demonstrated that the effect size of QTL associated with a trait played a role in the performance of GS and MAS models. For instance, for traits controlled by both large and moderate effect loci (FTFILD, FTRILD, SSFISD, and SSRISD), parametric model with known loci as fixed effect (FxRRBLUP) followed by MAS outperformed the rest of the GS models (RRBLUP, RKHS, and SVR). The use of known markers as fixed effects has been shown to increase prediction accuracy (Bernardo, 2014; Spindel et al., 2016) in parametric GS models. For traits that were controlled by moderate effect loci (FTFISD, FTRISD, MFISD, and MTRISD), our results showed that the two parametric GS models (FxRRBLUP and RRBLUP) and semi-parametric (RKHS) had similar prediction accuracy; however, FxRRBLUP had higher bias than RRBLUP and RKHS (**Figure S12**–**S13**). Furthermore, the performance of MAS in comparison to GS models in this study supported the fact that large effect loci are important influencers of MAS (Bernardo, 2008). For small breeding programs in developing countries, MAS might be a prudent choice over GS for traits controlled by large effects loci in cowpea since GS will require genotyping of more markers than MAS. The large effect loci identified in this study can be transferred to different breeding populations because they were identified in a MAGIC population with wide genetic background (Descalsota et al., 2018; Huynh et al., 2018). Our study thus demonstrates that prior knowledge of the genetic architecture of a trait can help make informed decision about the best GEB method to employ in breeding.

In summary, using the cowpea MAGIC population, our study identified both main QTL and two-way epistatic loci underlying FLT, MAT, and SS. These traits are oligogenic in genetic architecture with QTL effects ranging from small to large sizes. The effect size of the markers/QTL reported in this study may be upwardly biased due to the small size (*n* = 305) of the cowpea MAGIC population. Thus, studies with higher sample sizes (*n* > 1,000) will prove more accurate (Xu, 2003; King and Long, 2017). The identified QTL and their colocalized *a priori* genes will serve as stepping stone for future studies considering the molecular characterization of the genes underlying FLT, MAT, and SS in cowpea. Further, we demonstrated that prior knowledge of the genetic architecture of a trait can help make informed decision in GEB. Due to variations observed across photoperiod/environments for FLT, we will recommend the development of photoperiod insensitive lines in cowpea breeding. Also, given that some QTL were identified in specific environments, considerations should be given to field evaluation of mapping populations under contrasting environments that are representative of natural populations’ environmental conditions. In addition, the cowpea MAGIC population may not capture all the genetic variation available in cowpea for FLT, MAT, and SS because only eight founders were used for its development. Thus, some of our markers may not be well diagnostic in breeding populations that do not share close ancestry with the cowpea MAGIC founders. Despite this limitation, this study still provides technical details that can be part of considerations for GS and MAS in cowpea breeding.

## Data Availability

Publicly available datasets were analyzed in this study. This data can be found here: https://onlinelibrary.wiley.com/doi/full/10.1111/tpj.13827

## Author Contributions

MO obtained data from UCR; concept by MO and ZH; MO and ZH analyzed the data; MO, ZH, and PA wrote the manuscript. All authors read and approved the manuscript.

## Conflict of Interest Statement

The authors declare that the research was conducted in the absence of any commercial or financial relationships that could be construed as a potential conflict of interest.

## Supplementary Material

All the R scripts used for analyses in the study are available at: https://github.com/marcbios/Cowpea.git

Data S1 Candidate gene list

Data S2 Main effect QTL list

Data S3 Epistatic QTL list

Data S4 Genes that colocalized with main and epistatic QTL
