# Supplementary material for: Epistasis Detection and Modeling for Genomic Selection in Cowpea (Vigna unguiculata L. Walp.)
Source: Front Genet. 2019 Jul 30;10:677. doi: 10.3389/fgene.2019.00677 (PMC6682672; doi:10.3389/fgene.2019.00677)
Supplement: Supplementary file 5 [file Datasheet_1.pdf]

Supplementary figures

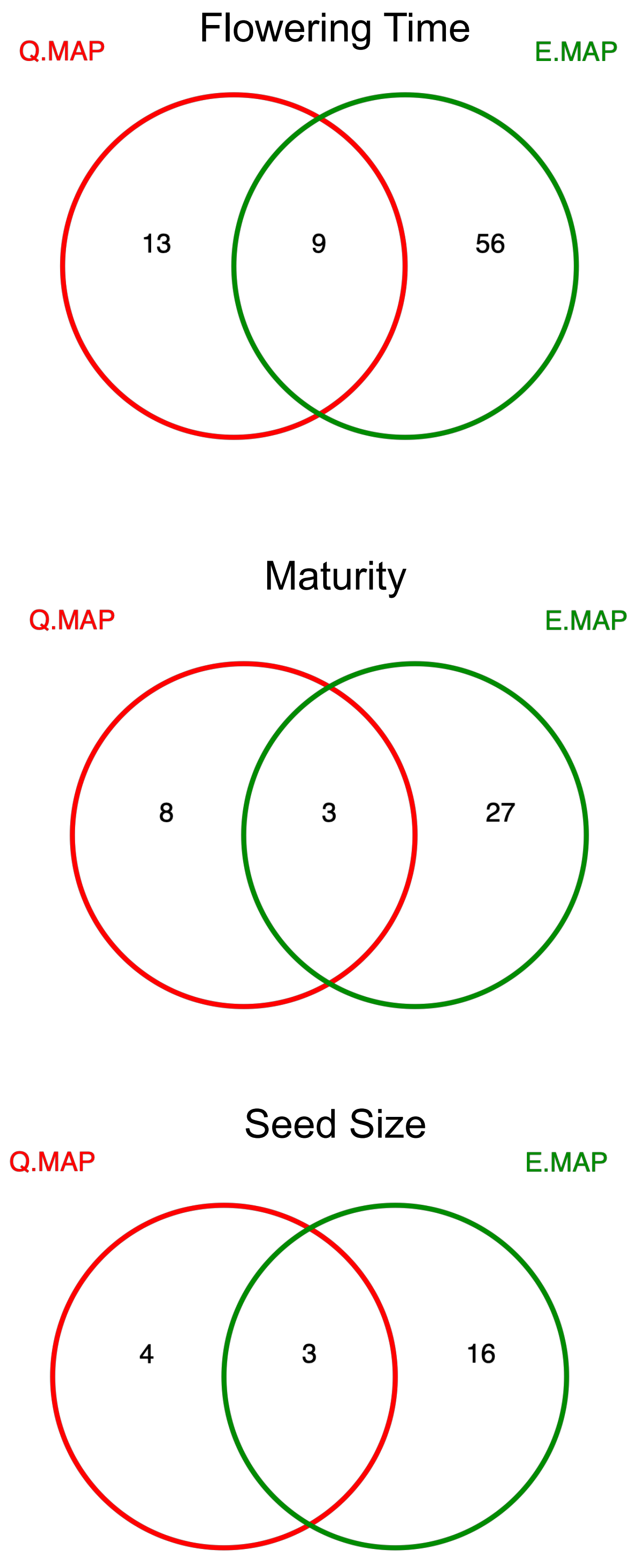

Figure S 1: Venn diagram of QTL overlap between main effect QTL mapping (Q.MAP) and epistasis mapping (E.MAP) for flowering time, maturity, and seed size.

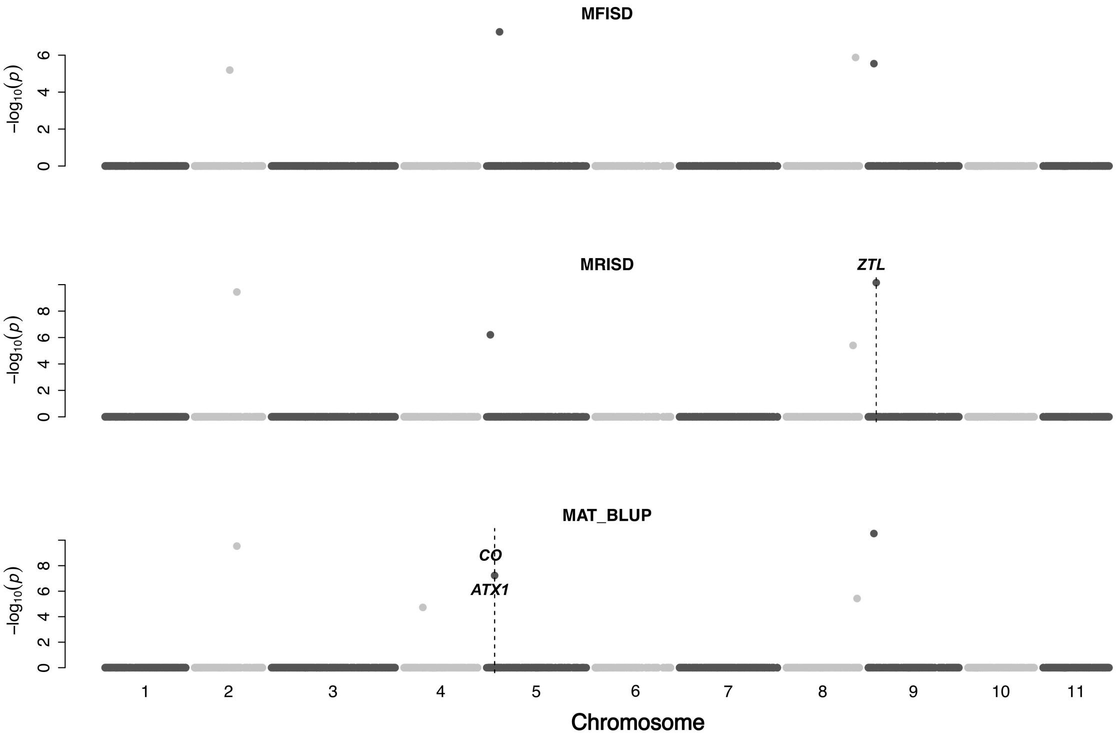

Figure S 2: QTL plot for maturity traits in the cowpea MAGIC population. QTL plots for maturity under full irrigation and short day (MFISD), maturity under restricted irrigation and short day (MRISD), and BLUPs of environments (MAT_BLUP). The chromosome numbers are located on the x-axis and the negative log of the *P*-values on the y-axis. The genetic position of the colocalization between QTL and *a priori* genes are indicated by broken vertical lines. The texts displayed on the vertical broken lines are the names of *a priori genes*.

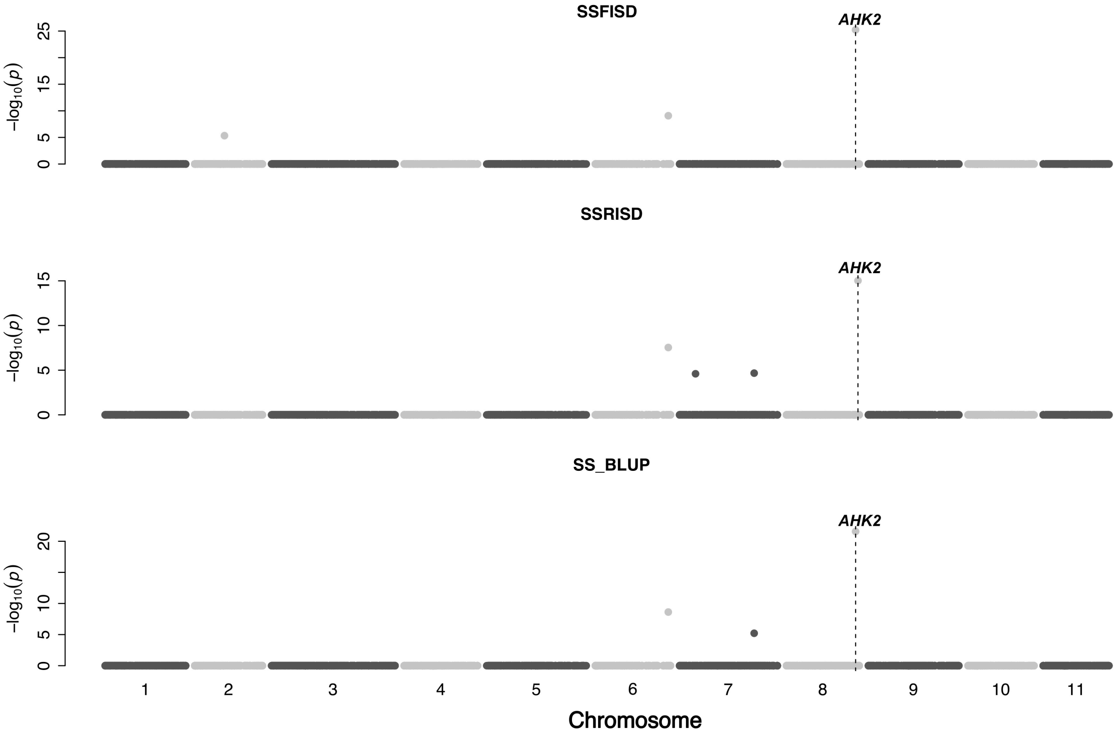

Figure S 3: QTL plot for seed size traits in the cowpea MAGIC population.QTL plots for maturity under full irrigation and short day (SSFISD), maturity under restricted irrigation and short day (SSRISD), and BLUPs of environments (SS_BLUP). The chromosome numbers are located on the x-axis and the

negative log of the *P*-values on the y-axis. The genetic position of the colocalization between QTL and *a priori* genes are indicated by broken vertical lines. The texts displayed on the vertical broken lines are the names of *a priori genes*.

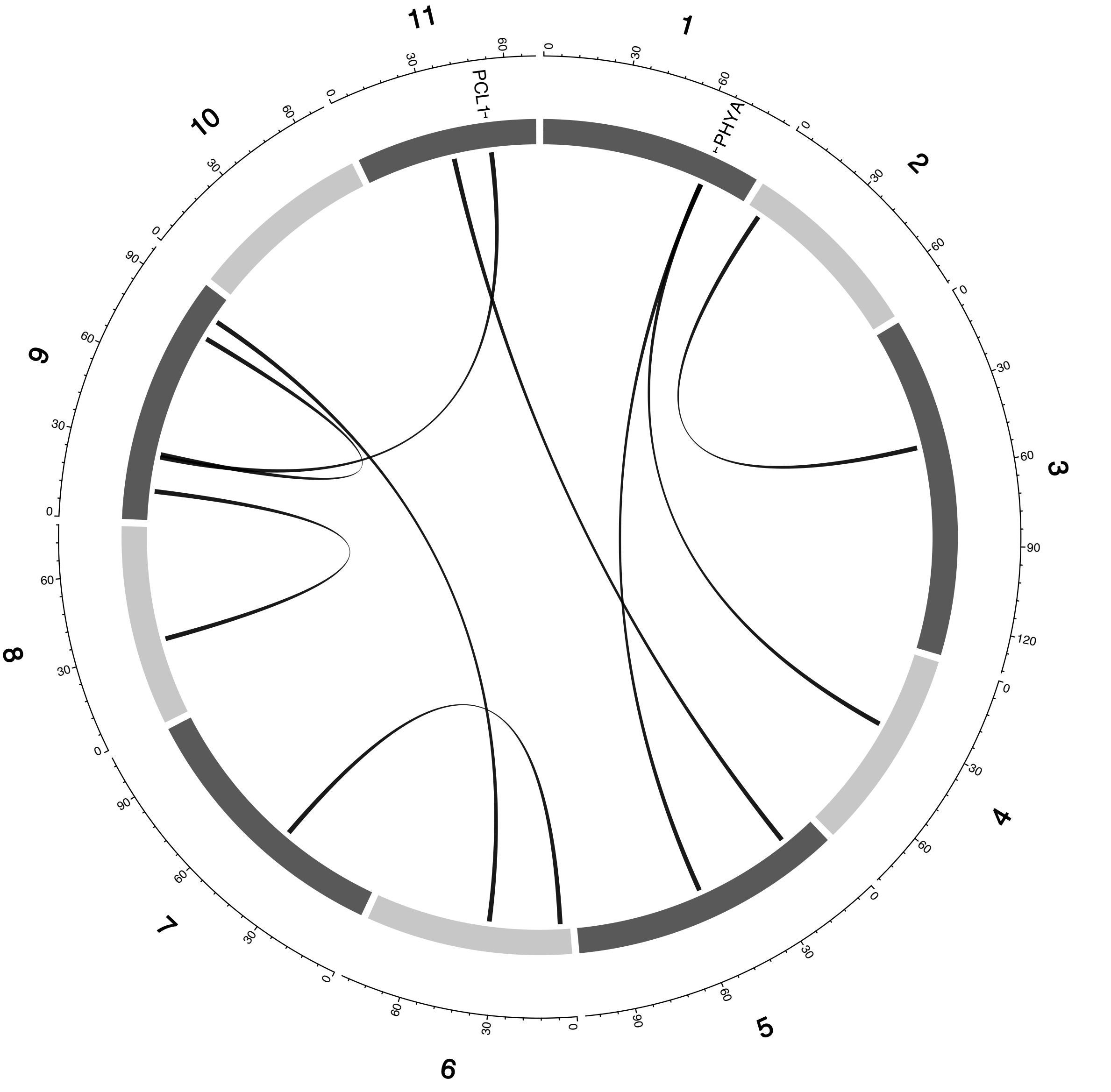

Figure S 4: Genetic map of the cowpea multiparent advanced generation inter-cross population (MAGIC) with pairwise interactions between epistatic QTL for FTFILD (Flowering time under full irrigation and long day). Chromosomes are shown in shades of gray, two-way interacting loci are connected with black solid lines, and colocalized *a priori* genes are texts between chromosomes and genetic map.

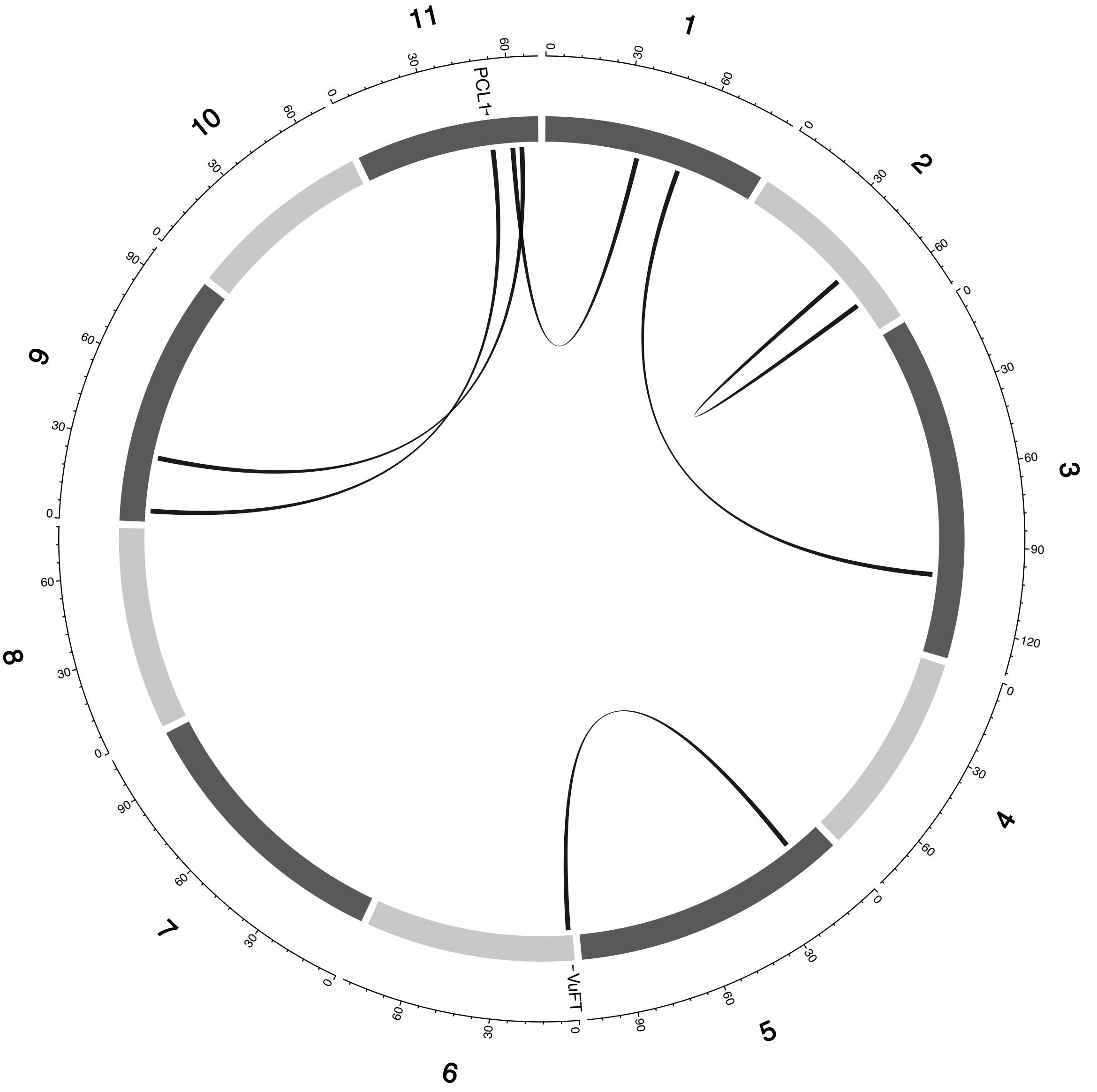

Figure S 5: Genetic map of the cowpea multiparent advanced generation inter-cross population (MAGIC) with pairwise interactions between epistatic QTL for FTRILD (Flowering time under restricted irrigation and long day). Chromosomes are shown in shades of gray, two-way interacting loci are connected with black solid lines, and colocalized a priori genes are texts between chromosomes and genetic map.

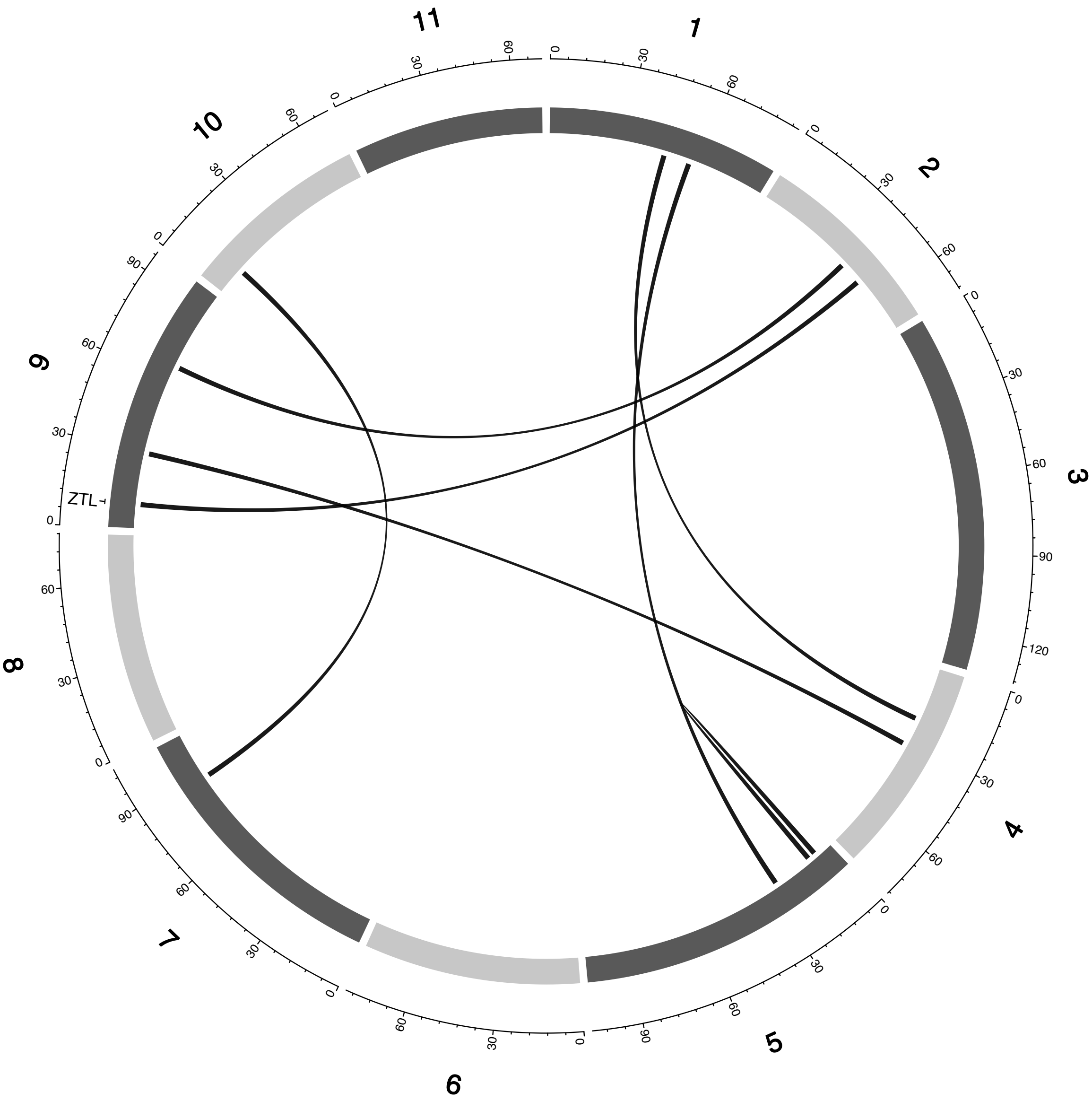

Figure S 6: Genetic map of the cowpea multiparent advanced generation inter-cross population (MAGIC) with pairwise interactions between epistatic QTL for FTFISD (Flowering time under full irrigation and short day). Chromosomes are shown in shades of gray, two-way interacting loci are connected with black solid lines, and colocalized a priori genes are texts between chromosomes and genetic map.

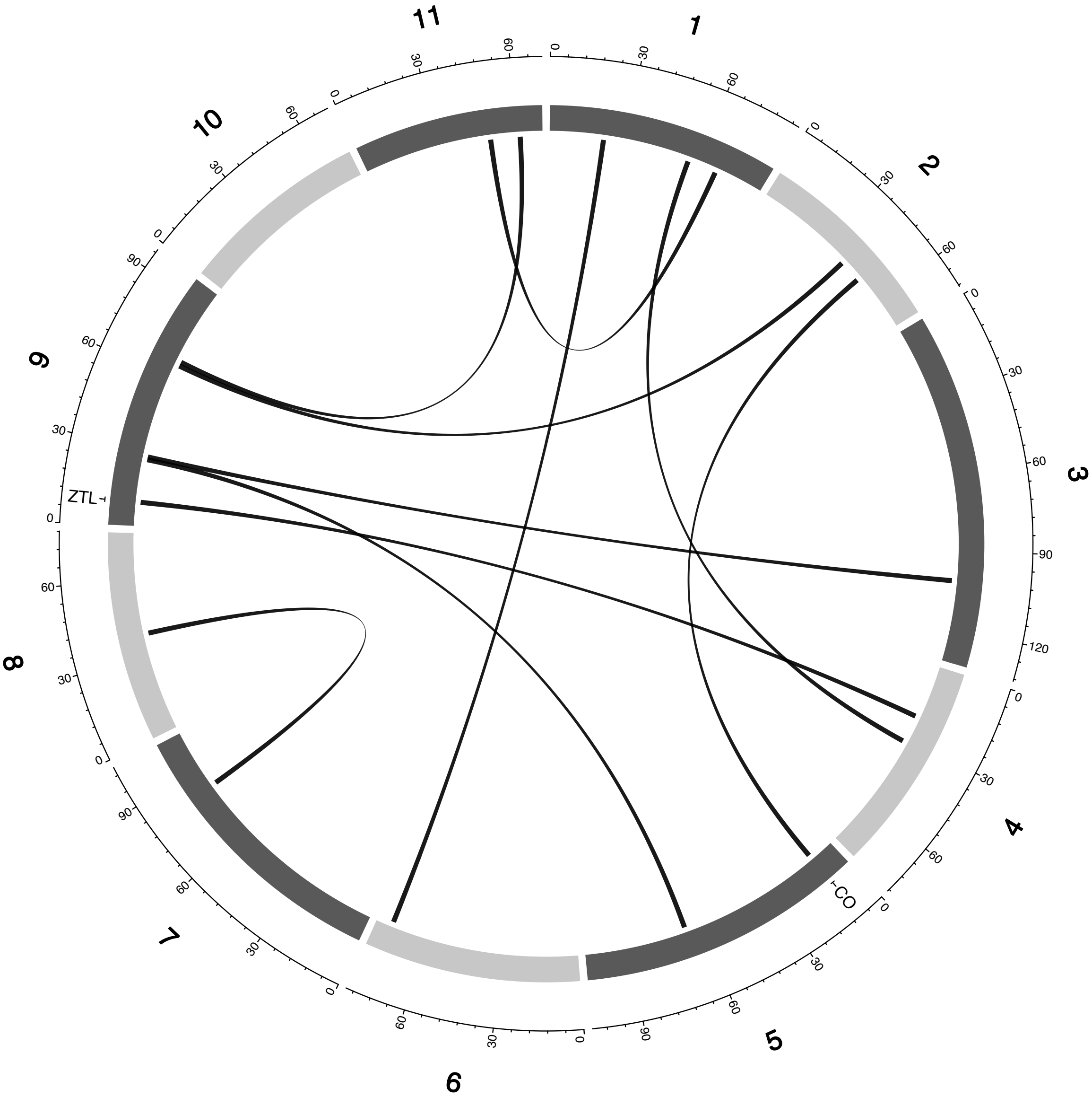

Figure S 7: Genetic map of the cowpea multiparent advanced generation inter-cross population (MAGIC) with pairwise interactions between epistatic QTL for FTRISD (Flowering time under restricted irrigation and short day). Chromosomes are shown in shades of gray, two-way interacting loci are connected with black solid lines, and colocalized a priori genes are texts between chromosomes and genetic map.

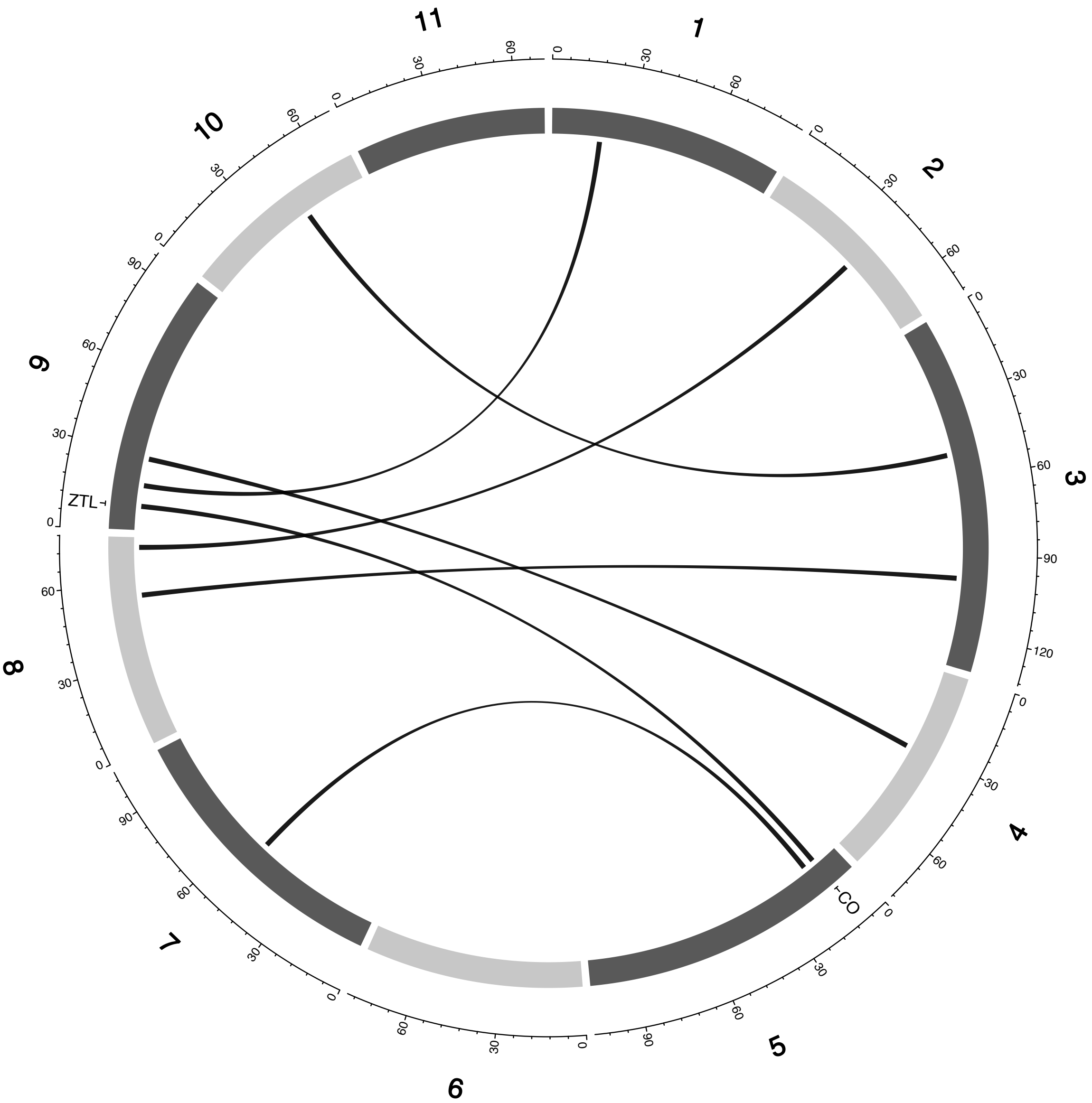

Figure S 8: Genetic map of the cowpea multiparent advanced generation inter-cross population (MAGIC) with pairwise interactions between epistatic QTL for MFISD (Maturity under full irrigation and short day). Chromosomes are shown in shades of gray, two-way interacting loci are connected with black solid lines, and colocalized a priori genes are texts between chromosomes and genetic map.

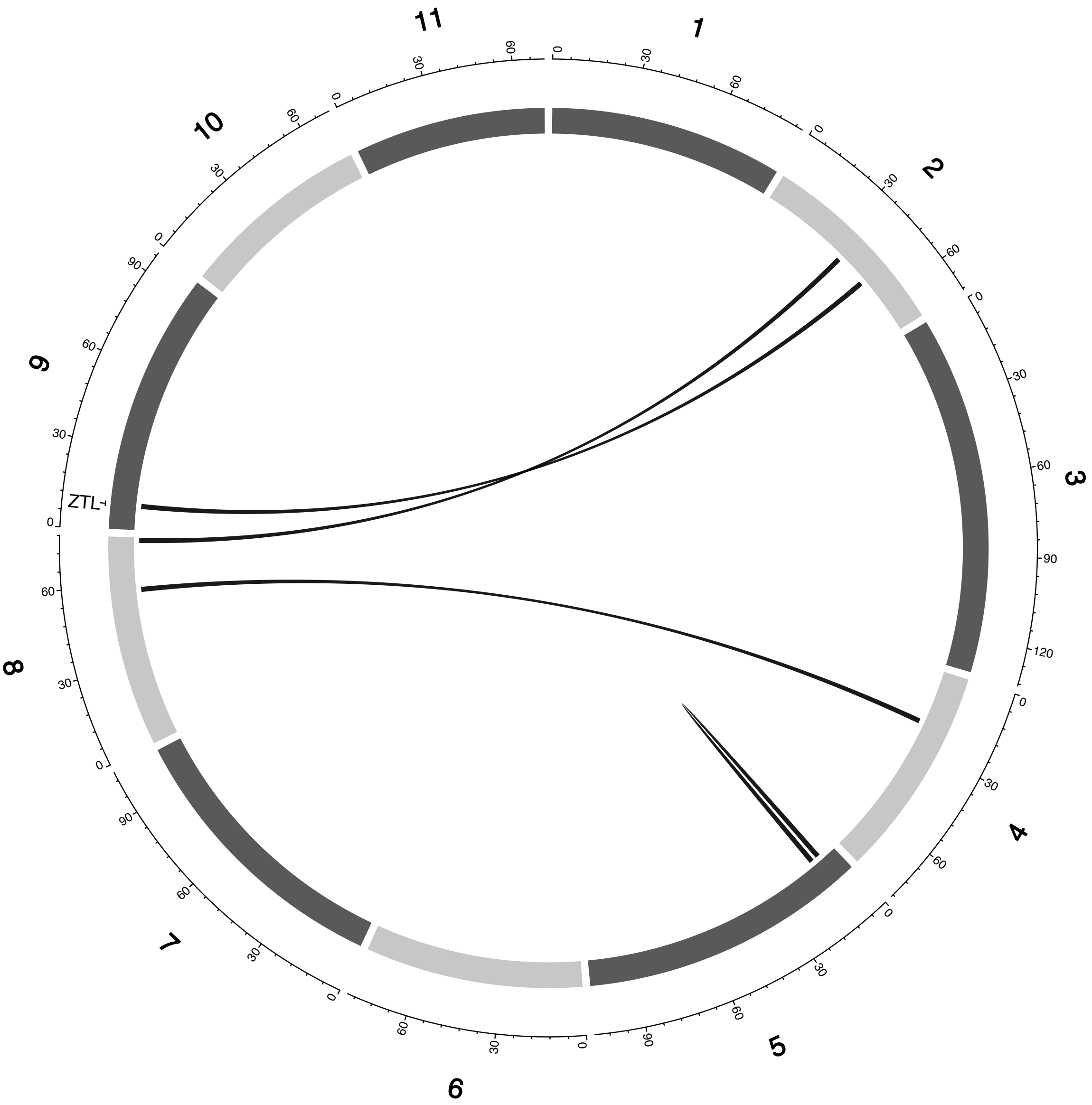

Figure S 9: Genetic map of the cowpea multiparent advanced generation inter-cross population (MAGIC) with pairwise interactions between epistatic QTL for MRISD (Maturity under restricted irrigation and short day). Chromosomes are shown in shades of gray, two-way interacting loci are connected with black solid lines, and colocalized a priori genes are texts between chromosomes and genetic map.

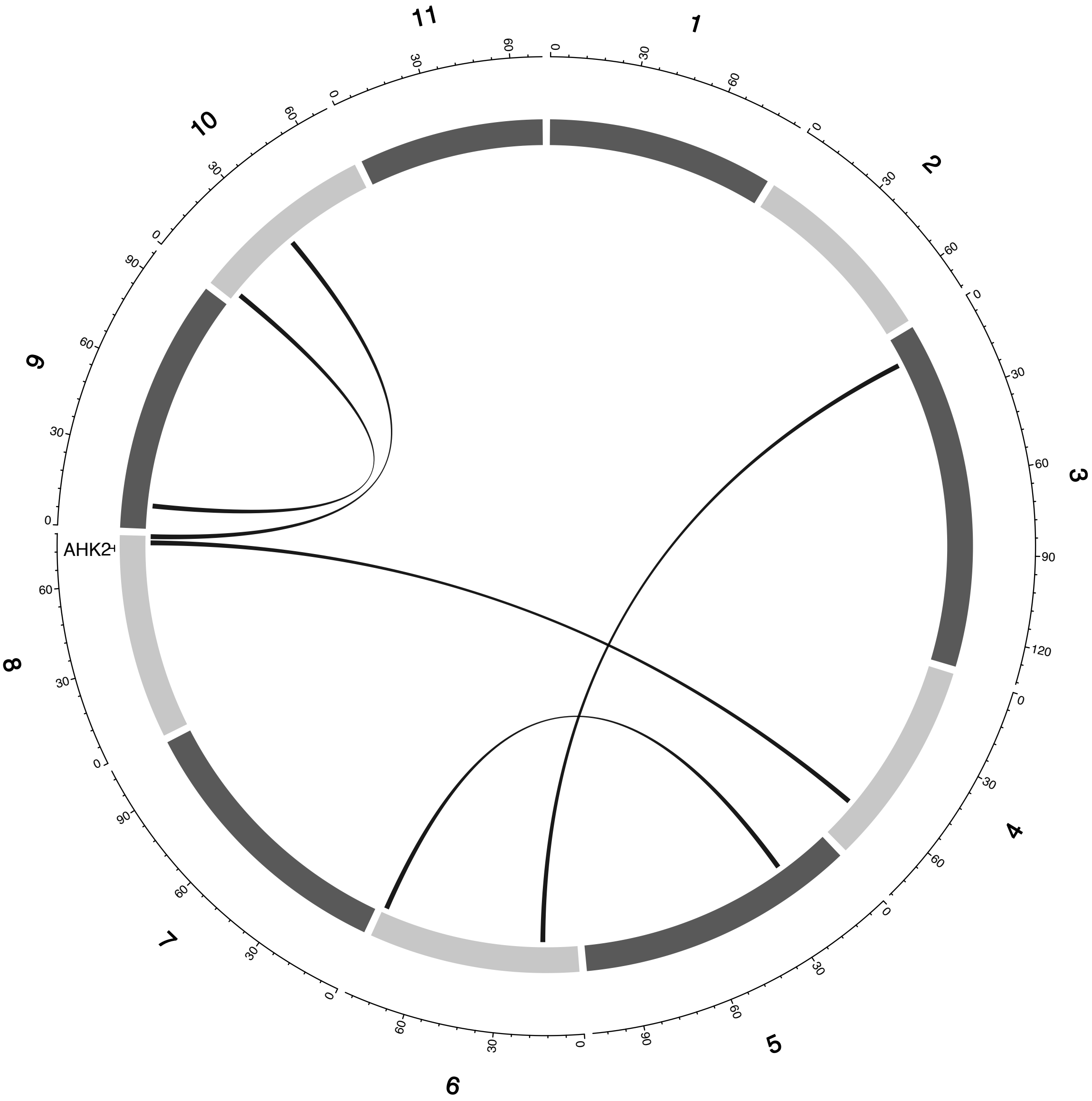

Figure S 10: Genetic map of the cowpea multiparent advanced generation inter-cross population (MAGIC) with pairwise interactions between epistatic QTL for SSFISD (Seed Size under full irrigation and short day). Chromosomes are shown in shades of gray, two-way interacting loci are connected with black solid lines, and colocalized a priori genes are texts between chromosomes and genetic map.

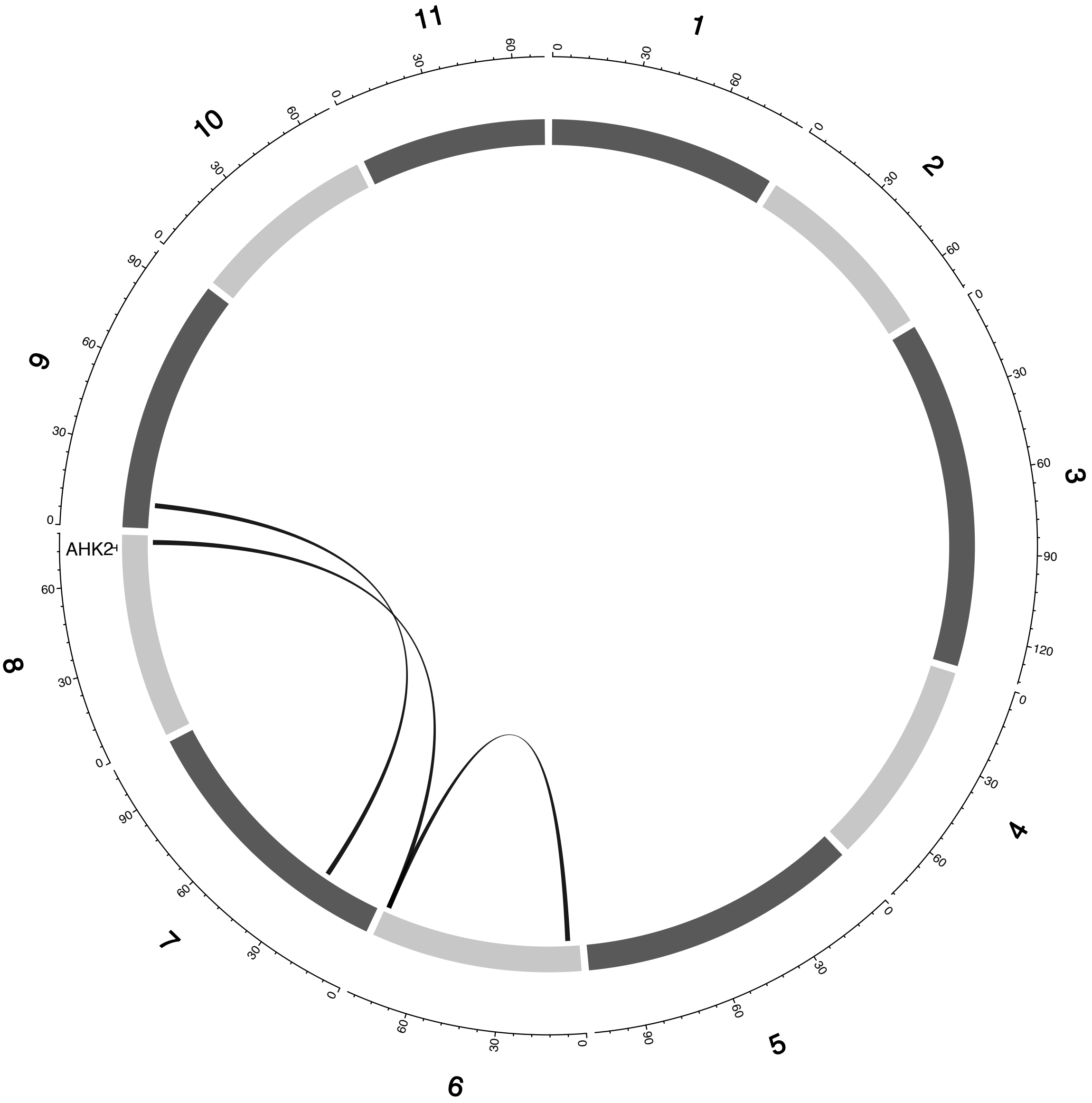

Figure S 11: Genetic map of the cowpea multiparent advanced generation inter-cross population (MAGIC) with pairwise interactions between epistatic QTL for SSRISD (Seed size under restricted irrigation and short day). Chromosomes are shown in shades of gray, two-way interacting loci are connected with black solid lines, and colocalized a priori genes are texts between chromosomes and genetic map.

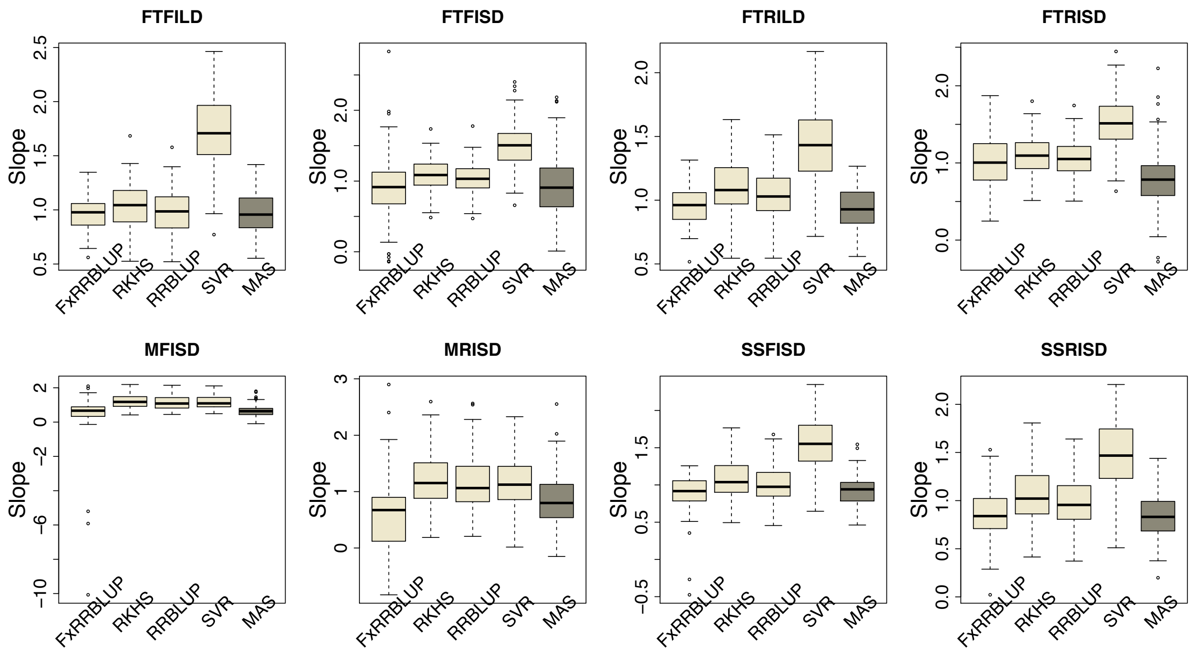

Figure S 12: Comparison of Slope of regression between observed and predicted trait values across GS and MAS models. Boxplots in each panel showed the distribution of slope values across 100 cycles for FxRRBLUP (Ridge Regression Best Linear Unbiased Prediction: Parametric model with fixed effects), RKHS (Reproducing Kernel Hilbert Space; Semi-Parametric model), RRBLUP (Ridge Regression Best Linear Unbiased Prediction: Parametric model with no fixed effects), SVR (Support Vector Regression: Non-Parametric model), and MAS (Marker Assisted Selection) for flowering time under full irrigation and long day (FTFILD), flowering time under restricted irrigation and long day (FTRILD), flowering time under full irrigation and short day (FTFISD), flowering time under restricted irrigation and short day (FTRISD), maturity under full irrigation and short day (MFISD), maturity under restricted irrigation and short day, seed size under full irrigation and short day (SSFISD), and seed size under restricted irrigation and short day (SSRISD).

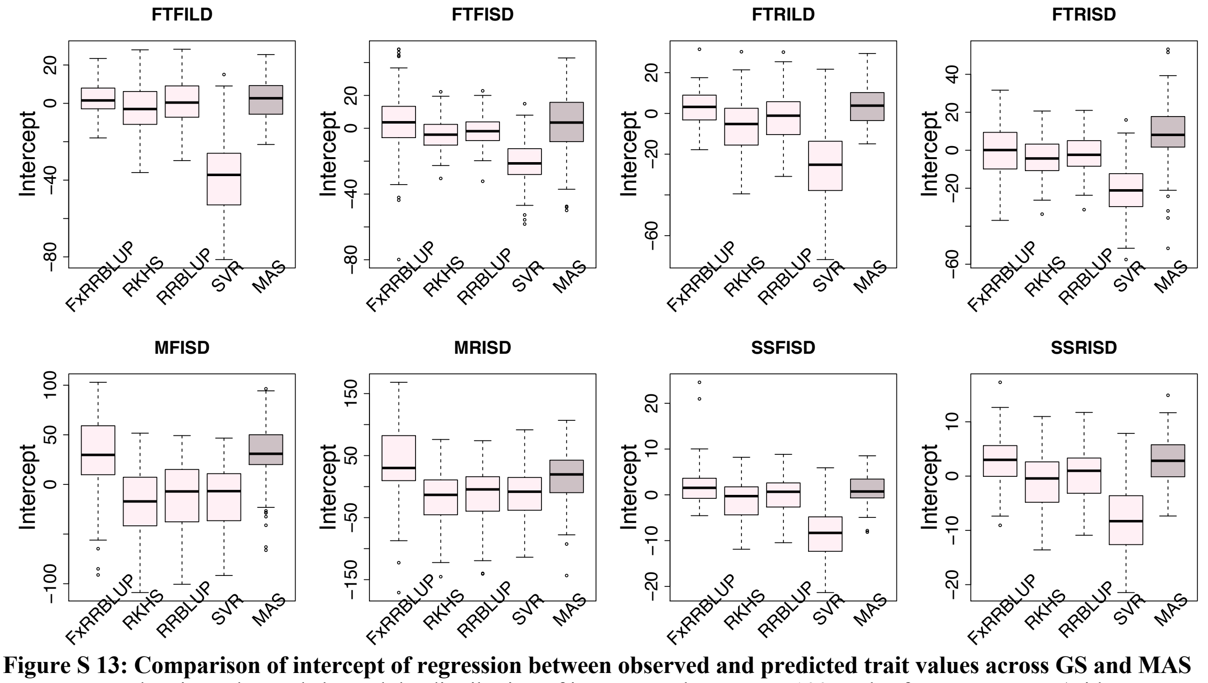

Figure S 13: Comparison of intercept of regression between observed and predicted trait values across GS and MAS models. Boxplots in each panel showed the distribution of intercept values across 100 cycles for FxRRBLUP (Ridge Regression Best Linear Unbiased Prediction: Parametric model with fixed effects), RKHS (Reproducing Kernel Hilbert Space; Semi-Parametric model), RRBLUP (Ridge Regression Best Linear Unbiased Prediction: Parametric model with no fixed effects), SVR (Support Vector Regression: Non-Parametric model), and MAS (Marker Assisted Selection) for flowering time under full irrigation and long day (FTFILD), flowering time under restricted irrigation and long day (FTRILD), flowering time under full irrigation and short day (FTFISD), flowering time under restricted irrigation and short

day (FTRISD), maturity under full irrigation and short day (MFISD), maturity under restricted irrigation and short day, seed size under full irrigation and short day (SSFISD), and seed size under restricted irrigation and short day (SSRISD).

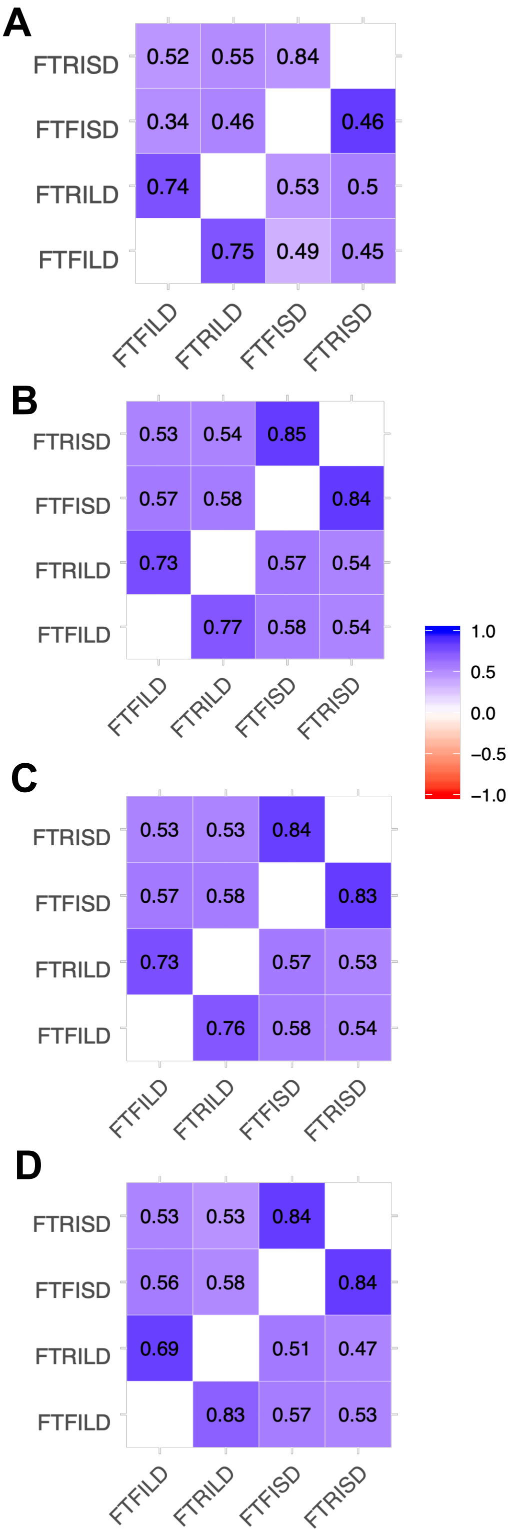

Figure S 14: Environment by environment prediction values across GS models. Boxplots in each panel showed the distribution of intercept values across 100 cycles for (A) FxRRBLUP (Ridge Regression Best Linear Unbiased Prediction: Parametric model with fixed effects), (B) RKHS (Reproducing Kernel Hilbert Space; Semi-Parametric model), (C) RRBLUP (Ridge Regression Best Linear Unbiased Prediction: Parametric model with no fixed effects), and (D) SVR (Support Vector Regression: Non-Parametric model) for flowering time under full irrigation and long day (FTFILD), flowering time under restricted irrigation and long day (FTRILD), flowering time under full irrigation and short day (FTFISD), flowering time under restricted irrigation and short day (FTRISD)

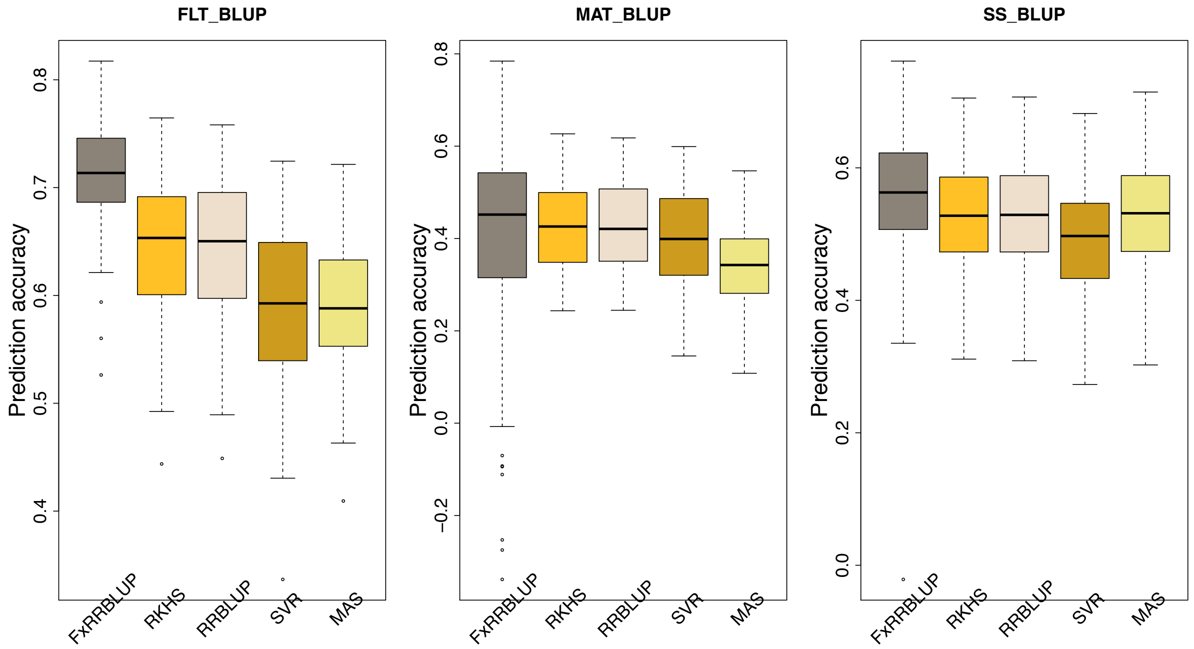

Figure S 15: Comparison of prediction accuracy across GS and MAS models. Boxplots in each panel showed the distribution of prediction accuracy values across 100 cycles for FxRRBLUP (Ridge Regression Best Linear Unbiased Prediction: Parametric model with fixed effects), RKHS (Reproducing Kernel Hilbert Space; Semi-Parametric model), RRBLUP (Ridge Regression Best Linear Unbiased Prediction: Parametric model with no fixed effects), SVR (Support Vector Regression: Non-Parametric model), and MAS (Marker Assisted Selection) for flowering time BLUP (FLT_BLUP), maturity BLUP (MAT_BLUP), seed size BLUP (SS_BLUP).

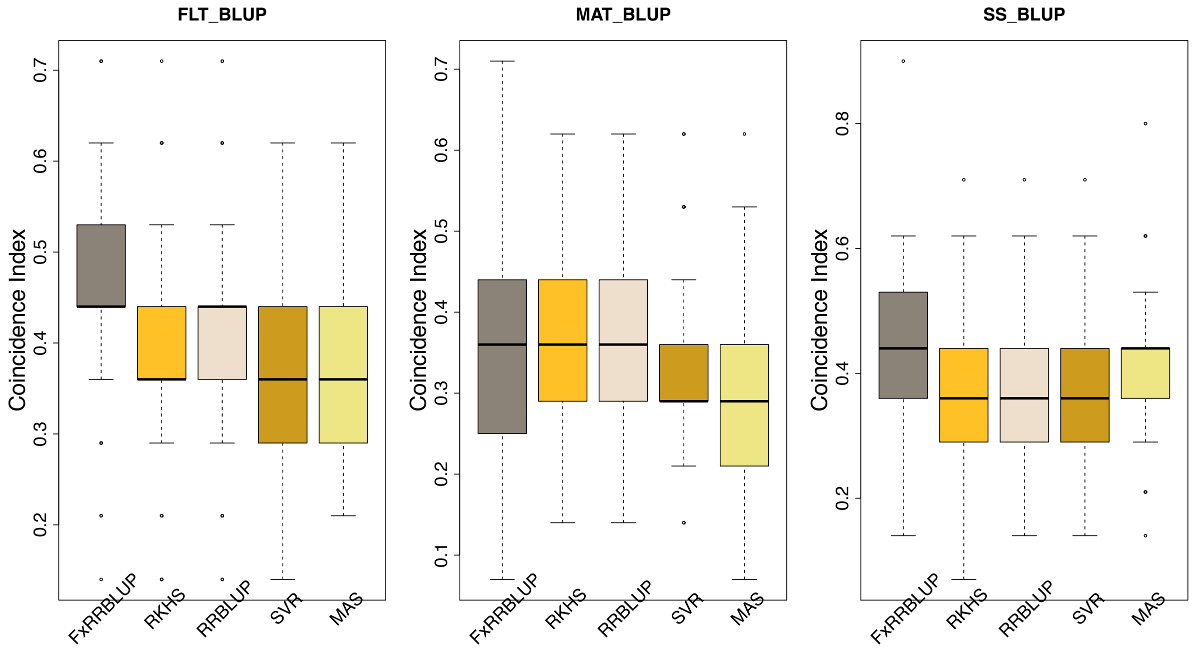

Figure S 16: Comparison of coincidence index across GS and MAS models. Boxplots in each panel showed the distribution of coincidence index values across 100 cycles for FxRRBLUP (Ridge Regression Best Linear Unbiased Prediction: Parametric model with fixed effects), RKHS (Reproducing Kernel Hilbert Space; Semi-Parametric model), RRBLUP (Ridge Regression Best Linear Unbiased Prediction: Parametric model with no fixed effects), SVR (Support Vector Regression: Non-Parametric model), and MAS (Marker Assisted Selection) for flowering time BLUP (FLT_BLUP), maturity BLUP (MAT_BLUP), seed size BLUP (SS_BLUP).

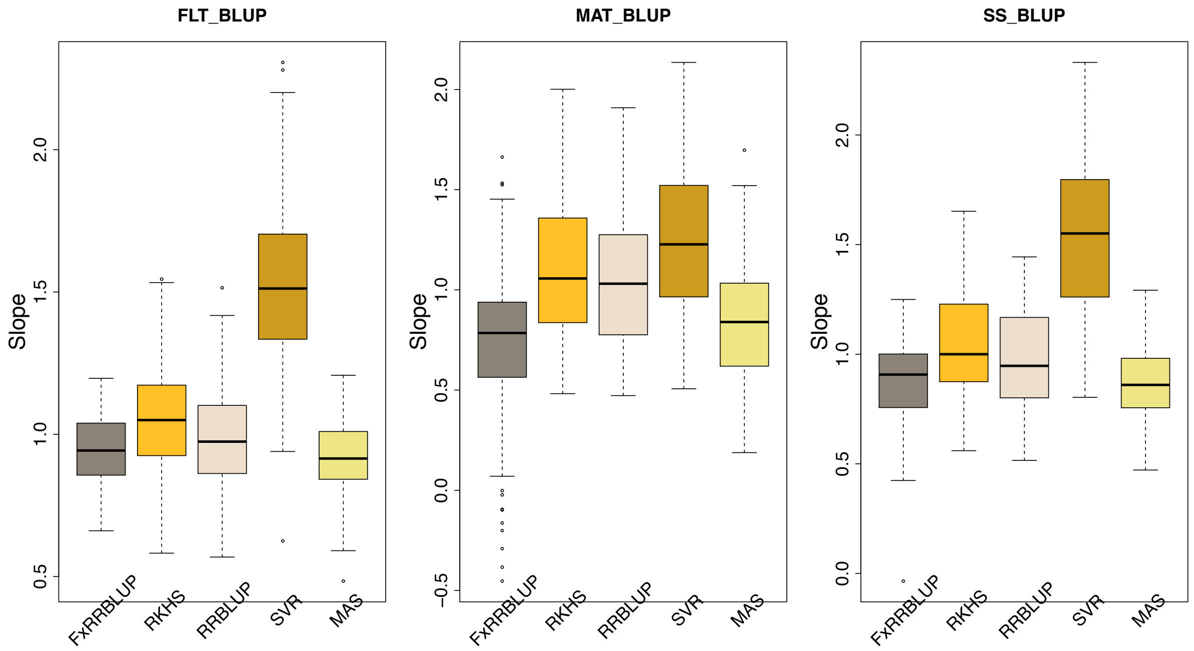

Figure S 17: Comparison of slope values across GS and MAS models. Boxplots in each panel showed the distribution of slope values across 100 cycles for FxRRBLUP (Ridge Regression Best Linear Unbiased Prediction: Parametric model with fixed effects), RKHS (Reproducing Kernel Hilbert Space; Semi-Parametric model), RRBLUP (Ridge Regression Best Linear Unbiased Prediction: Parametric model with no fixed effects), SVR (Support Vector Regression: Non-Parametric model), and MAS (Marker Assisted Selection) for flowering time BLUP (FLT_BLUP), maturity BLUP (MAT_BLUP), seed size BLUP (SS_BLUP).

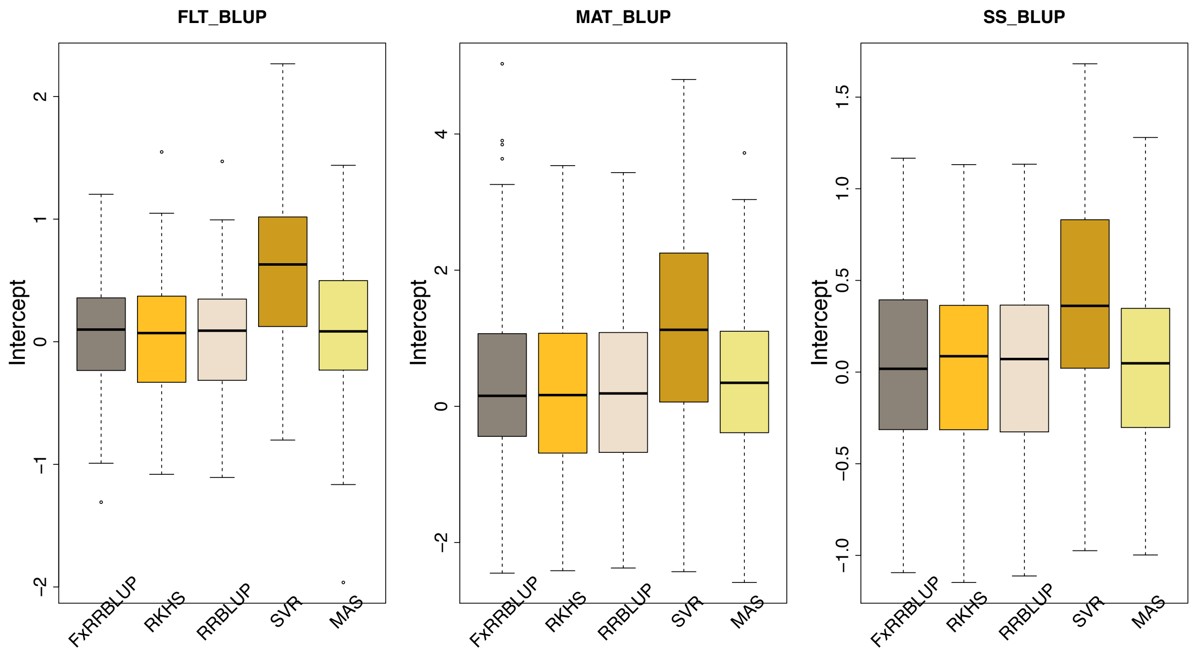

Figure S 18: Comparison of intercept values across GS and MAS models. Boxplots in each panel showed the distribution of intercept values across 100 cycles for FxRRBLUP (Ridge Regression Best Linear Unbiased Prediction: Parametric model with fixed effects), RKHS (Reproducing Kernel Hilbert Space; Semi-Parametric model), RRBLUP (Ridge Regression Best Linear Unbiased Prediction: Parametric model with no fixed effects), SVR (Support Vector Regression: Non-Parametric model), and MAS (Marker Assisted Selection) for flowering time BLUP (FLT_BLUP), maturity BLUP (MAT_BLUP), seed size BLUP (SS_BLUP).

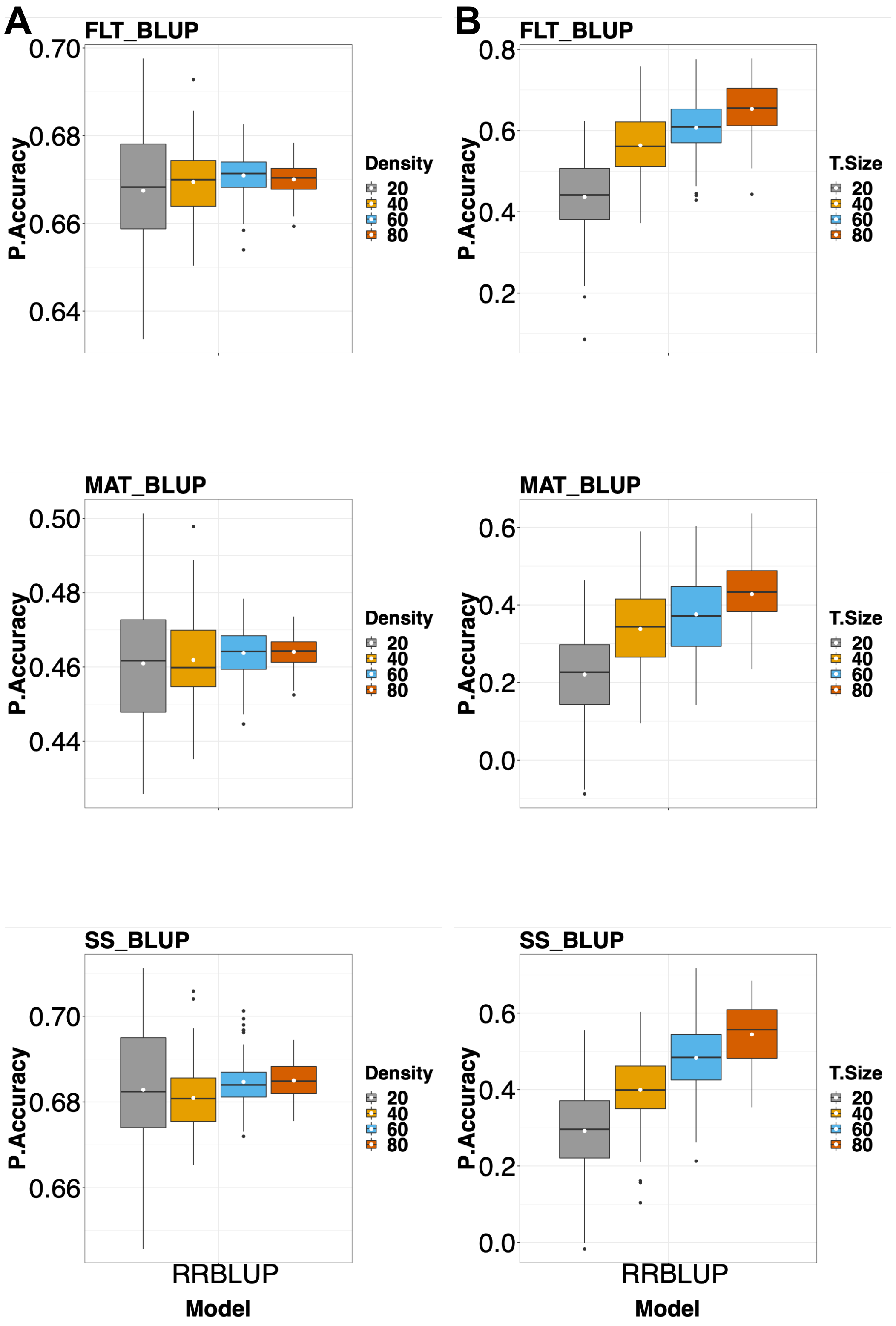

Figure S 19: The effect of marker density and training population size on prediction accuracy. (A) Boxplots showing comparison among different marker densities (20%, 40%, 60%, and 80%). (B) Boxplots showing comparison among different training population sizes (20%, 40%, 60%, and 80%).
