# Supplementary material for: Epistasis Detection and Modeling for Genomic Selection in Cowpea (Vigna unguiculata L. Walp.)
Source: Front Genet. 2019 Jul 30;10:677. doi: 10.3389/fgene.2019.00677 (PMC6682672; doi:10.3389/fgene.2019.00677)
Supplement: Supplementary file 6 [file Datasheet_2.pdf]

Supplementary Tables

Table S 1: Environment by Environment correlation for flowering time.

Traits	FTFILD	FTRILD	FTFISD	FTRISD
**FTFILD**	-	0.76	0.50	0.44
**FTRILD**		-	0.57	0.52
**FTFISD**			-	0.86
**FTRISD**				-
**Heritability (h^2)**	0.41	0.42	0.48	0.46

Flowering time under full irrigation and long day (FTFILD), flowering time under restricted irrigation and long day (FTRILD), flowering time under full irrigation and short day (FTFISD), flowering time under restricted irrigation and short day (FTRISD)

Table S 2: Environment by environment correlation for maturity and seed size.

Traits	MFISD	MRISD	SSFISD	SSRISD
**Heritability (h^2)**	0.30	0.21	0.47	0.39
**MFISD**	-	0.64	-	-
**SSFISD**			-	0.80

Maturity under full irrigation and short day (MFISD), maturity under restricted irrigation and short day (MRISD), seed size under full irrigation and short day (SSFISD), and seed size under restricted irrigation and short day (SSRISD)

Table S 3: QTL identified by stepwise regression explaining at least 6% of phenotypic variation.

Trait	QTL	Chr.	Pos.	PVE	ADE	MAF
FLT_BLUP	qVu9:24.77	9	24.7747	24.2	3.1	0.28
FLT_BLUP	qVu9:5.85	9	5.8471	13.0	2.1	0.4
FLT_BLUP	qVu11:49.06	11	49.0616	8.9	1.7	0.36
FLT_BLUP	qVu5:8.5	5	8.5038	7.8	1.6	0.48
FLT_BLUP	qVu4:29	4	29.0011	7.1	1.5	0.49
FTFILD	qVu9:24.77	9	24.7747	25.0	7	0.28
FTFILD	qVu9:22.65	9	22.648	19.5	5.6	0.49
FTFILD	qVu11:49.06	11	49.0616	10.9	4.4	0.36
FTFISD	qVu9:8.37	9	8.367	10.5	1.5	0.35
FTFISD	qVu4:31.3	4	31.2954	8.4	1.3	0.47
FTFISD	qVu5:8.5	5	8.5038	7.9	1.9	0.13
FTFISD	qVu4:20.34	4	20.3441	7.5	1.7	0.14
FTFISD	qVu2:40.37	2	40.3707	7.1	1.2	0.37
FTRILD	qVu9:23.36	9	23.3558	27.5	6.9	0.29
FTRILD	qVu5:8.5	5	8.5038	7.3	3.3	0.48
FTRILD	qVu11:49.06	11	49.0616	7.0	3.3	0.36
FTRISD	qVu4:19.99	4	19.9946	10.6	2.2	0.15
FTRISD	qVu1:55.11	1	55.111	8.2	1.4	0.47
FTRISD	qVu9:8.37	9	8.367	8.0	1.4	0.35
FTRISD	qVu4:31.3	4	31.2954	6.8	1.3	0.47
FTRISD	qVu5:3.9	5	3.8966	6.8	1.3	0.46
MAT_BLUP	qVu2:45.2	2	45.203	9.5	3.3	0.31
MAT_BLUP	qVu9:5.85	9	5.8471	8.6	3	0.38
MAT_BLUP	qVu4:19.99	4	19.9946	6.0	3.5	0.15
MFISD	qVu5:13.76	5	13.7582	8.2	3.5	0.35
MFISD	qVu9:5.85	9	5.8471	6.6	3	0.49
MRISD	qVu2:45.2	2	45.203	9.6	6.1	0.31
MRISD	qVu9:8.37	9	8.367	8.6	5.5	0.39
SS_BLUP	qVu8:74.29	8	74.2918	25.2	2.1	0.25
SS_BLUP	qVu6:78.35	6	78.3467	10.7	1.2	0.49
SSFISD	qVu8:74.21	8	74.2124	29.3	2.5	0.24
SSFISD	qVu6:78.35	6	78.3467	9.2	1.3	0.49
SSRISD	qVu8:76.81	8	76.8132	19.7	2.4	0.23
SSRISD	qVu6:78.35	6	78.3467	10.0	1.5	0.49

Quantitative trait loci (QTL), Chromosome (Chr.), Position (Pos. in centimorgan), Additive effect (ADE), Phenotypic variation explained (PVE), and Minor allele frequency (MAF). Flowering time BLUP (FLT_BLUP), maturity BLUP (MAT_BLUP), seed size BLUP (SS_BLUP), flowering time under full irrigation and long day (FTFILD), flowering time under restricted irrigation and long day (FTRILD), flowering time under full irrigation and short day (FTFISD), flowering time under restricted irrigation and short day (FTRISD), maturity under full irrigation and short day (MFISD), maturity under restricted irrigation and short day (MRISD), seed size under full irrigation and short day (SSFISD), and seed size under restricted irrigation and short day (SSRISD)

Table S 4: Epistatic QTL identified by SPAEML and their effect sizes.

Trait	QTL1	ADE1	MAF1	QTL2	ADE2	MAF2
FLT_BLUP	qVu9:25.39	3.1	0.28	qVu11:62.84	1.9	0.14
FLT_BLUP	qVu9:5.86	2.1	0.39	qVu11:42.83	1.8	0.14
FLT_BLUP	qVu5:12.79	1.7	0.34	qVu6:78.36	0.5	0.47
FLT_BLUP	qVu4:31.3	1.3	0.48	qVu6:1.47	1.6	0.15
FLT_BLUP	qVu1:66.57	1.6	0.12	qVu9:26.8	1.5	0.26
FLT_BLUP	qVu4:30.21	0.2	0.44	qVu7:45.81	0.4	0.43
FTFILD	qVu9:25.39	7	0.28	qVu11:50.94	4.6	0.26
FTFILD	qVu5:12.79	2.9	0.34	qVu11:35.28	1.6	0.40
FTFILD	qVu1:66.38	3.3	0.22	qVu4:31.03	2.3	0.47
FTFILD	qVu1:66.57	4.3	0.12	qVu5:52.97	0.9	0.37
FTFILD	qVu6:32.5	1.2	0.12	qVu9:86.49	1.1	0.34
FTFISD	qVu4:31.3	1.2	0.48	qVu9:28.65	0.8	0.41
FTFISD	qVu1:55.11	1.2	0.37	qVu5:25.01	0.4	0.10
FTFISD	qVu2:48.05	0.9	0.30	qVu9:8.37	1.4	0.39
FTFISD	qVu7:84.88	0.6	0.38	qVu10:10.07	0.8	0.36
FTFISD	qVu5:5.81	0.6	0.16	qVu5:8.91	1	0.49
FTRILD	qVu9:25.39	6.6	0.28	qVu11:62.84	3.8	0.14
FTRILD	qVu5:12.79	3.7	0.34	qVu6:0.68	2.4	0.39
FTRISD	qVu4:20.34	2.1	0.14	qVu9:8.37	1.3	0.39
FTRISD	qVu1:54.81	1.3	0.37	qVu4:31.3	1.2	0.48
FTRISD	qVu1:66.38	1.2	0.22	qVu11:49.06	1.1	0.39
FTRISD	qVu7:80.11	0.3	0.47	qVu8:37.41	0.6	0.48
MAT_BLUP	qVu2:48.05	2.8	0.30	qVu9:8.37	3	0.39
MFISD	qVu5:12.79	3.4	0.34	qVu7:50.14	2	0.27
MFISD	qVu2:39.29	2.4	0.36	qVu8:73.15	2.4	0.34
MFISD	qVu5:8.5	2.8	0.19	qVu9:8.37	2.8	0.39
MRISD	qVu2:48.05	5.3	0.30	qVu9:8.37	5.5	0.39
MRISD	qVu2:35.19	5.4	0.21	qVu8:75.88	4.8	0.24
SS_BLUP	qVu6:78.36	1.1	0.47	qVu7:18.1	0.4	0.11
SS_BLUP	qVu6:78.35	0.8	0.13	qVu8:74.29	2.1	0.25
SSFISD	qVu4:62.75	0.8	0.10	qVu8:74.29	2.5	0.25
SSFISD	qVu5:20.54	0.6	0.11	qVu6:78.36	1.2	0.47
SSRISD	qVu6:78.35	1	0.13	qVu8:74.29	2.2	0.25
SSRISD	qVu6:3.67	0.8	0.12	qVu6:78.36	1.4	0.47

Quantitative trait loci (QTL), Linkage group (LG), Position (Pos. in centimorgan), Additive effect (ADE), and Minor allele frequency (MAF). Flowering time BLUP (FLT_BLUP), maturity BLUP (MAT_BLUP), seed size BLUP (SS_BLUP), flowering time under full irrigation and long day (FTFILD), flowering time under restricted irrigation and long day (FTRILD), flowering time under full irrigation and short day (FTFISD), flowering time under restricted irrigation and short day (FTRISD), maturity under full irrigation and short day (MFISD), maturity under restricted irrigation and short day (MRISD), seed size under full irrigation and short day (SSFISD), and seed size under restricted irrigation and short day (SSRISD)

Table S 5: Mean and standard deviation of prediction accuracy across GS and MAS models.

Trait	FxRRBLUP	RKHS	RRBLUP	SVR	MAS
FT_BLUP	0.71±0.05	0.65±0.07	0.65±0.07	0.59±0.08	0.59±0.06
FTFILD	0.68±0.05	0.55±0.08	0.55±0.08	0.54±0.08	0.64±0.06
FTFISD	0.56±0.20	0.59±0.07	0.59±0.07	0.55±0.08	0.33±0.11
FTRILD	0.68±0.07	0.58±0.08	0.58±0.07	0.50±0.08	0.61±0.08
FTRISD	0.58±0.17	0.58±0.07	0.58±0.07	0.53±0.08	0.25±0.10
MT_BLUP	0.40±0.23	0.42±0.09	0.42±0.09	0.40±0.10	0.33±0.09
MFISD	0.30±0.20	0.39±0.09	0.39±0.09	0.36±0.09	0.20±0.10
MRISD	0.25±0.29	0.37±0.11	0.37±0.11	0.34±0.12	0.30±0.10
SS_BLUP	0.56±0.10	0.52±0.08	0.53±0.08	0.49±0.09	0.53±0.09
SSFISD	0.58±0.14	0.54±0.08	0.54±0.08	0.51±0.09	0.57±0.08
SSRISD	0.50±0.11	0.45±0.08	0.45±0.08	0.43±0.09	0.47±0.10

Flowering time BLUP (FLT_BLUP), maturity BLUP (MAT_BLUP), seed size BLUP (SS_BLUP), flowering time under full irrigation and long day (FTFILD), flowering time under restricted irrigation and long day (FTRILD), flowering time under full irrigation and short day (FTFISD), flowering time under restricted irrigation and short day (FTRISD), maturity under full irrigation and short day (MFISD), maturity under restricted irrigation and short day (MRISD), seed size under full irrigation and short day (SSFISD), and seed size under restricted irrigation and short day (SSRISD). FxRRBLUP (Ridge Regression Best Linear Unbiased Prediction: Parametric model with fixed effects). RKHS (Reproducing Kernel Hilbert Space; Semi-Parametric model), RRBLUP (Ridge Regression Best Linear Unbiased Prediction: Parametric model with no fixed effects), and SVR (Support Vector Regression: Non-Parametric model)

Table S 6: Mean and standard deviation of coincidence index of GS and MAS models.

Trait	FxRRBLUP	RKHS	RRBLUP	SVR	MAS
FLT_BLUP	0.47±0.11	0.40±0.11	0.42±0.11	0.37±0.10	0.37±0.09
FTFILD	0.49±0.09	0.37±0.10	0.37±0.10	0.35±0.11	0.45±0.10
FTFISD	0.43±0.16	0.44±0.10	0.44±0.10	0.42±0.09	0.30±0.11
FTRILD	0.40±0.09	0.36±0.10	0.35±0.10	0.28±0.10	0.37±0.10
FTRISD	0.45±0.15	0.43±0.09	0.42±0.10	0.42±0.10	0.26±0.09
MT_BLUP	0.34±0.15	0.35±0.09	0.36±0.10	0.33±0.11	0.27±0.10
MFISD	0.32±0.12	0.30±0.09	0.30±0.10	0.31±0.09	0.26±0.09
MRISD	0.31±0.18	0.34±0.10	0.35±0.10	0.33±0.10	0.33±0.10
SS_BLUP	0.44±0.11	0.37±0.11	0.36±0.10	0.36±0.10	0.42±0.11
SSFISD	0.48±0.11	0.44±0.09	0.44±0.09	0.43±0.09	0.46±0.10
SSRISD	0.42±0.11	0.36±0.11	0.37±0.11	0.36±0.10	0.40±0.10

Flowering time BLUP (FLT_BLUP), maturity BLUP (MAT_BLUP), seed size BLUP (SS_BLUP), flowering time under full irrigation and long day (FTFILD), flowering time under restricted irrigation and long day (FTRILD), flowering time under full irrigation and short day (FTFISD), flowering time under restricted irrigation and short day (FTRISD), maturity under full irrigation and short day (MFISD), maturity under restricted irrigation and short day (MRISD), seed size under full irrigation and short day (SSFISD), and seed size under restricted irrigation and short day (SSRISD). FxRRBLUP (Ridge Regression Best Linear Unbiased Prediction: Parametric model with fixed effects). RKHS (Reproducing Kernel Hilbert Space; Semi-Parametric model), RRBLUP (Ridge Regression Best Linear Unbiased Prediction: Parametric model with no fixed effects), and SVR (Support Vector Regression: Non-Parametric model)

Table S 7: Ranking of performance of GS models for each trait in a particular environment based on prediction n accuracy and coincidence index.

Trait	FxRRBLUP	RKHS	RRBLUP	SVR	MAS
FLT_BLUP	1	3	2	4	4
FTFILD	1	3	3	4	2
FTFISD	2	1	1	3	4
FTRILD	1	3	4	5	2
FTRISD	1	2	3	3	4
MT_BLUP	3	2	1	4	5
MFISD	1	3	3	2	4
MRISD	4	2	1	3	3
SS_BLUP	1	3	4	4	2
SSFISD	1	3	3	4	2
SSRISD	1	4	3	4	2

Flowering time BLUP (FLT_BLUP), maturity BLUP (MAT_BLUP), seed size BLUP (SS_BLUP), flowering time under full irrigation and long day (FTFILD), flowering time under restricted irrigation and long day (FTRILD), flowering time under full irrigation and short day (FTFISD), flowering time under restricted irrigation and short day (FTRISD), maturity under full irrigation and short day (MFISD), maturity under restricted irrigation and short day (MRISD), seed size under full irrigation and short day (SSFISD), and seed size under restricted irrigation and short day (SSRISD). FxRRBLUP (Ridge Regression Best Linear Unbiased Prediction: Parametric model with fixed effects). RKHS (Reproducing Kernel Hilbert Space; Semi-Parametric model), RRBLUP (Ridge Regression Best Linear Unbiased Prediction: Parametric model with no fixed effects), and SVR (Support Vector Regression: Non-Parametric model)
